# Elephant *TP53*-*RETROGENE 9* induces transcription-independent apoptosis at the mitochondria

**DOI:** 10.1038/s41420-023-01348-7

**Published:** 2023-02-16

**Authors:** Aidan J. Preston, Aaron Rogers, Miranda Sharp, Gareth Mitchell, Cristhian Toruno, Brayden B. Barney, Lauren N. Donovan, Journey Bly, Ryan Kennington, Emily Payne, Anthony Iovino, Gabriela Furukawa, Rosann Robinson, Bahar Shamloo, Matthew Buccilli, Rachel Anders, Sarah Eckstein, Elizabeth A. Fedak, Tanner Wright, Carlo C. Maley, Wendy K. Kiso, Dennis Schmitt, David Malkin, Joshua D. Schiffman, Lisa M. Abegglen

**Affiliations:** 1 Huntsman Cancer Institute, University of Utah, Salt Lake City, UT USA; 2 Scripps Green Hospital and Scripps Clinic, La Jolla, CA USA; 3 Recursion Pharmaceuticals, Salt Lake City, UT USA; 4 Duke Psychiatry and Behavioral Sciences, Duke University School of Medicine, Durham, NC USA; 5 Department of Mathematics, University of Utah, Salt Lake City, UT USA; 6 Department of Epigenetics and Molecular Carcinogenesis, The University of Texas MD Anderson Cancer Center, Houston, TX USA; 7 Biodesign Institute, School of Life Sciences, and Arizona Cancer Evolution Center, Arizona State University, Tempe, AZ USA; 8 Colossal Biosciences, Dallas, TX USA; 9 Department of Animal Science, William H. Darr College of Agriculture, Missouri State University, Springfield, MO USA; 10 Division of Haematology/Oncology, The Hospital for Sick Children; Department of Pediatrics, University of Toronto, Toronto, ON Canada; 11 Division of Pediatric Hematology/Oncology, Department of Pediatrics, University of Utah, Salt Lake City, UT USA; 12 Peel Therapeutics, Salt Lake City, UT USA

**Keywords:** Apoptosis, Cancer prevention, Mechanisms of disease

## Abstract

Approximately 20 *TP53* retrogenes exist in the African and Asian elephant genomes (*Loxodonta Africana, Elephas Maximus*) in addition to a conserved *TP53* gene that encodes a full-length protein. Elephant *TP53-RETROGENE 9* (*TP53-R9*) encodes a p53 protein (p53-R9) that is truncated in the middle of the canonical DNA binding domain. This C-terminally truncated p53 retrogene protein lacks the nuclear localization signals and oligomerization domain of its full-length counterpart. When expressed in human osteosarcoma cells (U2OS), p53-R9 binds to Tid1, the chaperone protein responsible for mitochondrial translocation of human p53 in response to cellular stress. Tid1 expression is required for p53-R9-induced apoptosis. At the mitochondria, p53-R9 binds to the pro-apoptotic BCL-2 family member Bax, which leads to caspase activation, cytochrome c release, and cell death. Our data show, for the first time, that expression of this truncated elephant p53 retrogene protein induces apoptosis in human cancer cells. Understanding the molecular mechanism by which the additional elephant *TP53* retrogenes function may provide evolutionary insight that can be utilized for the development of therapeutics to treat human cancers.

## Introduction

African and Asian elephants evolved ~20 additional *TP53* genes in their genome [1–3]. One *TP53* allele is orthologous to other mammalian *TP53* genes, whereas the other copies of elephant *TP53* are retrogenes [1, 2]. The expansion of *TP53* copy number in the elephant genome may have occurred randomly or due to evolutionary pressure; elucidating the function of an elephant *TP53* retrogene would provide evolutionary insight into the potential cancer-protective role of these retrogenes.

We previously reported that upon exposure to DNA damage, peripheral blood lymphocytes and fibroblasts from elephants undergo significantly more apoptosis compared to the same cells from healthy humans, and healthy human cells undergo significantly more apoptosis than cells from humans with a germline variant in one *TP53* allele, i.e., Li-Fraumeni Syndrome [1]. The lower threshold for DNA-damage-induced apoptosis in non-malignant cells correlates with the number of *TP53* alleles. Elephants, with their natural cancer resistance, are exemplars of Peto’s Paradox—defined as the lack of correlation between lifespan, body size, and cancer risk across species [4]. With a substantially more massive body and only slightly shorter lifespan than humans, the cancer mortality rate of elephants is lower than in humans [1, 5]. A recent study of cancer prevalence in elephants under human care found no malignant tumors in African elephants (*N* = 35) [3]. Increased *TP53* copy number and enhanced *TP53* activity may be part of the elephant’s solution to Peto’s paradox, protecting them from increased oncogenic risk due to their body size and lifespan. Discovering the mechanisms of these evolved defenses may inform the development of therapeutics to treat or prevent human cancers [6].

**Fig. 1:**
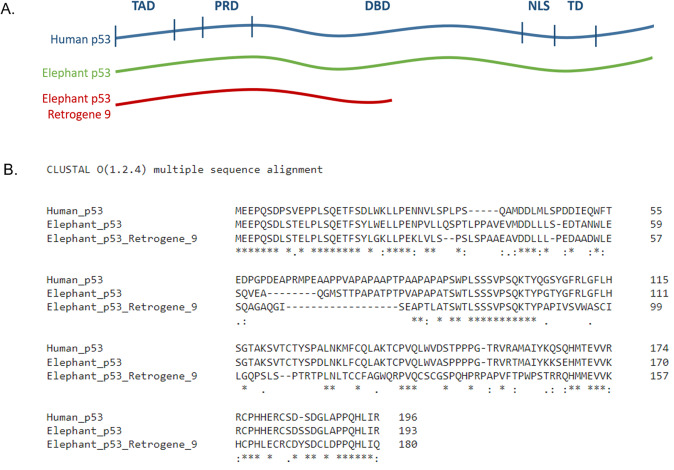
Elephant p53-Retrogene 9 encodes a truncated version of full-length elephant or human p53. **A** Cartoon of human p53, elephant p53, and elephant p53 retrogene 9, showing protein lengths and alignment of p53-R9 to known domains of human p53. TAD Transactivation Domain, PRD Proline-Rich Domain, DBD DNA Binding Domain, NLS Nuclear Localization Sequence, TD Tetramerization Domain. **B** Clustal multiple sequence alignment of human p53, elephant p53, and elephant-p53-retrogene 9 is shown up to the stop codon in p53-retrogene 9.

Of the ~20 *TP53* retrogenes in the African elephant genome, *TP53-RETROGENE 9* (*TP53-R9*) (GenBank KF715863, Ensembl ENSLAFG00000028299) was reported to be the most highly expressed in various Asian and African elephant tissues [2]. This gene encodes for p53-R9 protein, which has 180 amino acids and 54.1% pairwise identity when aligned to the full-length elephant p53 protein (Fig. 1A, B). Absent the carboxy terminus of the full-length p53 protein, p53-R9 lacks the residues necessary for canonical p53 tetramerization and predicted full DNA-binding potential [7, 8]. However, the amino terminus of human p53 has been shown to be sufficient to induce apoptosis, independent of transcriptional activity [9]. We hypothesized that the elephant retrogene might retain the ability to induce apoptosis, despite differences in amino acid sequences between the amino terminus of human p53 and p53-R9.

p53 participates in a transcription-independent apoptosis pathway after trafficking to the mitochondria [10]. While full-length human p53 activates Bak, Bax, and Bid and inhibits BCL-2, BCL-xL, and Mcl-1, C-terminally truncated human p53s of different lengths directly bind to Bak and BCL-xL [11–17]. Thus, it is possible that if p53-R9 induces cell death independent of full-length p53, that it may be acting through this secondary, transcription-independent mitochondrial pathway.

Here we characterize the apoptotic mechanism of p53-R9 in human *TP53-*deficient cancer cells. We establish that p53-R9 induces apoptosis with a potency comparable to equivalently truncated human p53. We show that p53-R9 interacts with chaperone protein Tid1 and traffics to the mitochondria where it induces cytochrome c release. We also demonstrate that p53-R9 induces cell death at the mitochondria via an interaction with pro-apoptotic protein Bax.

## Results

### Expression of elephant p53 retrogene 9 induces apoptosis of human cancer cells

Elephant lymphocytes and fibroblasts undergo more apoptosis than human lymphocytes and fibroblasts when treated with the same doses of ionizing radiation and doxorubicin, respectively [1]. To gauge whether p53 retrogenes play a role in the enhanced apoptotic phenotype of elephant cells, we aimed to determine if p53-R9 is capable of inducing apoptosis. We assessed the apoptotic capabilities of p53-R9 in human cancer cells due to an incomplete elephant genome assembly, a lack of immortalized elephant cell lines, and issues with cross-species specificity of antibodies that were developed to recognize human or mouse proteins.

**Fig. 2:**
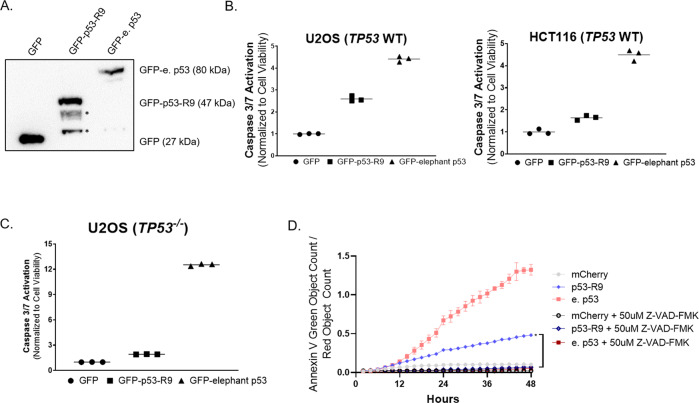
p53-R9 induces apoptosis of human cancer cells in the presence or absence of wild-type human *TP53*. **A** Immunoblot of U2OS (*TP53*^−/−^) cell lysates after transfection with green fluorescent protein (GFP)-tagged plasmids show expression of GFP, GFP-p53-R9, and GFP-e. p53 at predicted sizes. Blot was probed with antibody to GFP. Bands lower than 47 kDa in p53-R9 lane are likely cleavage products or non-specific bands, indicated by *. **B** Human cancer cells (U2OS and HCT116) were transfected and sorted for expression of the fluorescent protein GFP, then analyzed for Caspase 3/7 activity using the Promega Caspase-Glo 3/7 assay, values were normalized to cell viability (Cell Titer Glo). *p* < 0.01 for both p53-R9 and elephant p53 v GFP; One way ANOVA v GFP with Dunnett’s multiple comparison test and Dunnet’s correction. **C** U2OS *TP53*^−/−^ cells were transfected with plasmids encoding GFP-tagged proteins and sorted for expression of GFP. Caspase 3/7 activity was measured using the Promega Caspase-Glo 3/7 assay, values were normalized to cell viability (Cell Titer Glo). *p* < 0.0001 for both p53-R9 and elephant p53 v GFP; One way ANOVA with Dunnett’s multiple comparison test. **D** U2OS *TP53*^−*/−*^ cells expressing mCherry were analyzed for quantity of Annexin V green positive cells. Area under the curve (AUC) was calculated for *N* = 3 experiments; Welch’s *t* test comparing AUCs, *p < 0.01 p53-R9 v p53-R9 + Z-VAD-FMK. Error bars represent SD of three images/well taken in three wells of a representative experiment.

We transfected various human cancer cell lines that express wild-type *TP53* with plasmids encoding p53-R9, tagged with GFP for sorting and quantified activation of Caspases 3/7. We confirmed expression of GFP, GFP-p53-R9, GFP-elephant p53, mCherry, mCherry-p53-R9, and mCherry-elephant p53 as proteins in cells by Western blot (Figs. 2A and SI1a). p53-R9 expression induces apoptosis in osteosarcoma (U2OS) and colorectal cancer cells (HCT116) (*p* < 0.01 for each cell line, One-way ANOVA with Dunnett’s correction of GFP v p53-R9 and GFP) (Fig. 2B). However, p53-R9 expression results in less caspase activation in all these tumor cells than expression of full-length elephant p53 protein (Fig. 2B). These results resemble previously published data showing that expression of a C-terminally truncated human p53 causes apoptosis, but less than the expression of the full human p53 protein [9]. We aimed to determine if p53-R9 induces a similar magnitude of apoptosis as truncated elephant and human p53, or if p53-R9 evolved additional apoptotic potential. To assess the p53-independent apoptotic capacity of p53-R9, we knocked out endogenous *TP53* from U2OS osteosarcoma cells with CRISPR/Cas9 (Fig. SI2a, b). We focused our study on osteosarcoma because *TP53* is mutated in over 70% of these tumors, and novel approaches to improve p53 activity could improve patient outcomes [18–21]. In addition, we generated *TP53* null U2OS cells, despite the availability of another *TP53* null osteosarcoma cell line (Saos-2), because isogenic cell lines allowed us to assess *TP53* dependence in the absence of other genomic alterations.

To determine if p53-R9 induces apoptosis independent of p53 expression, *TP53*^−/−^ U2OS cells were transfected with mCherry, mCherry-p53-R9, or mCherry-e. p53. p53-R9 induced apoptosis in *TP53*^−/−^ cells, indicating that apoptosis occurs independent of p53 expression (Fig. 2C). These results were confirmed in other *TP53*^−/−^ (SAOS-2 and HCT116) and *TP53* mutant (T98G) cell lines (Fig. SI1b).

We wanted to rule out the possibility that p53-R9 expression in cancer cells triggers the unfolded protein response (UPR), which results in apoptosis from endoplasmic reticulum stress due to the accumulation of unfolded proteins [22]. We did not observe an upregulation in biomarkers of UPR, including C/EBP Homologous Protein (CHOP) and Activating transcription factor 6 (ATF6-N), in cells expressing p53-R9 (Fig. SI3a). In addition, we did not detect an increase in Eukaryotic Translation Initiation Factor 2A (EIF2a) phosphorylation or the splicing of X-Box binding protein mRNA after p53-R9 expression (Fig. SI3a, b). These results suggest that p53-R9 induces apoptosis through a specific mechanism rather than being a non-specific result of over-expressed, unfolded protein accumulation.

To confirm that p53-R9 induces cell death specifically through caspase activation, we utilized the pan-caspase inhibitor Z-VAD-FMK. We first validated the efficacy of Z-VAD-FMK in our U2OS (*TP53*^−/−^) model by confirming that it inhibits staurosporine-induced apoptosis (Fig. SI4a, b). Next, we demonstrated that in the presence of 50 µM Z-VAD-FMK, neither p53-R9 nor full-length elephant p53 induces cell death over 2 days as quantified by Annexin V positivity (Fig. 2D). The complete abrogation of cell death in U2OS (*TP53*^−/−^) treated with Z-VAD-FMK further strengthened our hypothesis that p53-R9 is a pro-apoptotic protein.

**Fig. 3:**
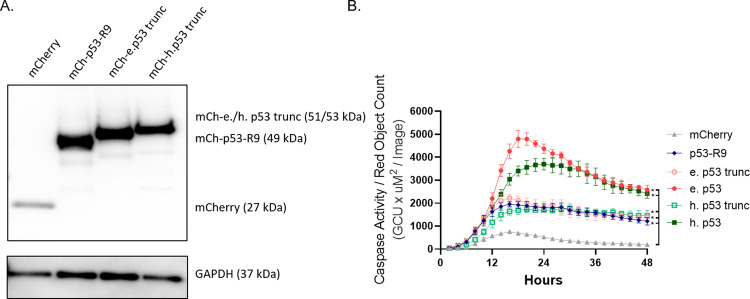
p53-R9 induces apoptosis similarly to truncated elephant and human p53. **A** U2OS *TP53*^−/−^ cells were transfected with mCherry, mCh-p53-R9, mCh-truncated elephant p53, mCh-elephant p53, mCh-truncated human p53, or mCh-human p53. Immunoblots were performed on cell lysates to confirm expression of proteins of the predicted sizes. Blots were probed with antibodies to mCherry and GAPDH. **B** Transfected and sorted U2OS *TP53*^*−/−*^ cells expressing mCherry or the indicated mCherry tagged proteins were analyzed for Caspase-3/7 activity (Caspase-3/7 green apoptosis assay reagent used with the Incucyte Live-Cell Imaging and Analysis System). Area under the curve (AUC) was calculated for *N* = 3 experiments. One-way ANOVA with Dunnett Correction compared each AUC with mCherry-only control: *p < 0.01 for p53-R9, truncated elephant and human p53 and full-length elephant and human p53 vs mCherry. One-way ANOVA with Dunnett Correction compared the AUC for truncated elephant and human p53 with p53-R9: *p* > 0.05. There was no significant difference between the apoptotic capacity of these proteins. Error bars represent SD of three images/well across three wells of a representative experiment.

Finally, to determine if p53-R9 induced a similar magnitude of apoptosis as truncated elephant and human p53, plasmids encoding truncated elephant (V194*) and human p53 (V197*) were designed to contain stop codons at the sites aligned with the stop codon of p53-R9 (Fig. 1B). mCherry fusion proteins of the predicted size were expressed in cells transfected with each construct (Fig. 3A) p53-R9 expression in U2OS (*TP53*^−/−^) induced similar cell death (caspase activity) compared to plasmids encoding truncated elephant and human p53 protein, each of which caused less cell death than their full-length protein counterparts (Fig. 3B). After observing similar caspase activation induced by the expression of p53-R9, we sought to understand the mechanism by which p53-R9 can induce apoptosis.

### p53-R9 does not upregulate common p53 target genes

**Fig. 4:**
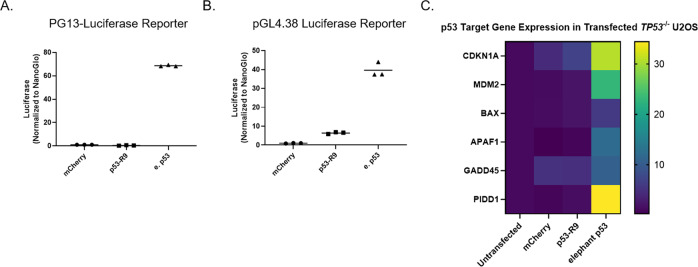
p53-R9 does not induce transcription of common p53 target genes. U2OS *TP53*^−/−^ cells were transfected with a luciferase reporter vector containing either the **A** PG13 human p53 response element or **B** pGL4.38 human p53 response elements. Controls included the control NanoGlo vector and the mCherry vector. Reporter activity was measured by luciferase expression and graphed. **A** p53-R9 did not induce significantly more luciferase expression than the mCherry control; One-way ANOVA with Dunnett’s correction, *p* > 0.05. Elephant p53 induced significantly more expression of luciferase than mCherry control; One-way ANOVA with Dunnett’s correction, *p* < 0.0001. **B** p53-R9 induced significantly more luciferase expression than the mCherry control; One-way ANOVA with Dunnett’s correction, *p* < 0.05. Elephant p53 induced significantly more expression of luciferase than mCherry control; One-way ANOVA with Dunnett’s correction, *p* < 0.0001. **C** Quantitative rt-PCR was performed and changes in mRNA expression of six canonical p53 target genes with mCherry, mCh-p53-R9, and mCh-elephant p53 expression in U2OS *TP53*^−/−^ cells relative to untransfected cells are represented by heat map.

While human p53 can trigger apoptosis through direct interactions with proteins at the mitochondria, it is primarily a transcription factor capable of inducing apoptosis by upregulating pro-apoptotic and downregulating anti-apoptotic genes in response to stress [23]. However, p53-R9 has a stop codon in the middle of the canonical DNA binding domain, resulting in a p53 protein that lacks both the tetramerization domain and the C-terminal nuclear localization sequences (Fig. 1A) [24]. We hypothesized that p53-R9 cannot transactivate p53 target genes because it lacks much of the DNA binding domain. To assess p53-R9’s ability to activate target genes, p53-R9 was transfected into U2OS *TP53*^−/−^ cells along with luciferase reporter plasmids with p53 response elements (PG13-Luc and pGL4.38) [25]. p53-R9 expression did not robustly activate luciferase expression from either reporter plasmid, which each contains different p53 response elements (Fig. 4A, B). There was a statistically significant, but small, difference in luciferase expression when p53-R9 was co-transfected with the PGL4.38 vector. To determine if this difference might indicate that p53-R9 could activate some p53 response elements, but not others, we also tested p53-R9’s ability to affect expression of known p53 target genes by qPCR. We observed no change in gene expression in U2OS *TP53*^−/−^ cells transfected with p53-R9, while elephant p53 transactivated these targets as expected (Fig. 4C). Although it remains possible that p53-R9 can transactivate p53 targets that we did not measure and/or noncanonical targets, these data support the hypothesis that p53-R9 functions through a transcription-independent apoptotic pathway, potentially though direct interaction at the mitochondria.

### p53-R9-induced apoptosis is associated with localization at the mitochondria

**Fig. 5:**
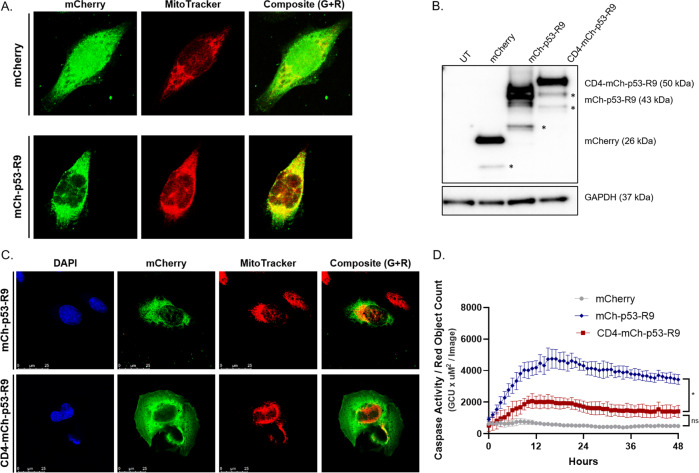
p53-R9 localizes at the mitochondria and forced localization at the cell surface significantly decreases apoptosis. **A** U2OS *TP53*^−/−^ cells were induced with doxycycline to express mCherry or mCh-p53-R9. Cells were stained with antibody to mCherry (green) and MitoTracker (red), then analyzed by confocal microscopy. Composite image shows both green (G) and red (R) signal. Colocalization of p53-R9 and mitotracker are shown in yellow. Colocalization of p53-R9 and MitoTracker are shown in yellow. **B** U2OS *TP53*^−/−^ cells were transfected to express mCherry, mCh-p53-R9, CD4-mCh-p53-R9, or untransfected. 24 h post-transfection, an immunoblot was performed on cell lysates. Blot was probed with antibodies to mCherry and GAPDH. Cleavage products or non-specific bands indicated by *. **C** CD4-mCh-p53-R9 localizes at the cell membrane. Confocal composite images of a z-stack of a single cell expressing mCh-p53-R9 or CD4-mCh-p53-R9. mCherry was visualized with antibody to mCherry (green), DAPI was used to visualize the nucleus (blue), and MitoTracker was used to visualize the mitochondria (red). Composite image shows both green (G) and red (R) signal. **D** U2OS *TP53*^*−/−*^ cells expressing mCherry, mCh-p53-R9, or CD4-mCh-p53-R9 were analyzed for Caspase-3/7 activity (Caspase-3/7 green apoptosis assay reagent used with the Incucyte Live-Cell Imaging and Analysis System). AUC was calculated for *N* = 3 experiments; Welch’s t-test of AUCs comparing p53-R9 to CD4-p53-R9, *p < 0.05. There is no significant difference between the apoptosis induced by CD4-mCh-p53-R9 and the mCherry control vector; Welch’s *t* test, ns *p* > 0.05. Error bars represent SD of three images/well taken in three wells of a representative experiment.

Upon cellular insult, cytoplasmic p53 can induce transcription-independent apoptosis by rapidly localizing to the mitochondria prior to cyctochrome c release and caspase activation [10, 26]. Therefore, we tested the hypothesis that p53-R9 induces apoptosis through a transcription-independent, mitochondrial mechanism. To better control the timing of expression of p53-R9, we generated a U2OS *TP53*^−/−^ cell line with tetracycline-inducible mCherry-tagged p53-R9 protein. A tetracycline-inducible mCherry expressing cell line was generated as a control and doxycycline-inducible expression of mCherry and mCherry-p53-R9 were confirmed by Western blot and microscopy (Fig. SI5a, b). After tetracycline induction and sorting for mCherry positivity, cells were treated with Mitotracker (red) to label the mitochondria and mCherry antibody (green) was used to detect expression of mCherry or mCherry-p53-R9. We observed co-expression of mCh-p53-R9 with Mitotracker (yellow), suggesting that p53-R9 traffics to the mitochondria (Fig. 5A). We also performed live-cell imaging to track p53-R9 expression within the cells, and we observed that p53-R9 protein associates with the mitochondria just prior to cell death (Movies 1–5).

To determine if localization of p53-R9 at the mitochondria is required for apoptosis induction, we designed a vector to target p53-R9 expression outside of the mitochondria. We tagged p53-R9 with the N-terminus of plasma membrane protein CD4 (UniProt Q13969), which includes the signal peptide that targets it to the cell surface. First, we confirmed that the fusion protein was expressed at the predicted size by Western blot in lysates from U2OS *TP53*^−/−^ transfected cells (Fig. 5B) Then, we confirmed that mCherry-CD4-p53-R9 localized to the cell surface and not the mitochondria of U2OS *TP53*^−/−^ cells by confocal microscopy (Fig. 5C, Movies 6–7). Preventing p53-R9 from trafficking to the mitochondria by targeting it to the plasma membrane significantly decreased caspase activation (Fig. 5D) (*p* < 0.05, Welch’s *t* test), suggesting that in the absence of full-length p53, p53-R9 induces apoptosis through a mitochondrial-mediated mechanism.

### p53-R9 binds chaperone Tid1 to localize at the mitochondria and apoptosis is dependent on Tid1 expression

**Fig. 6:**
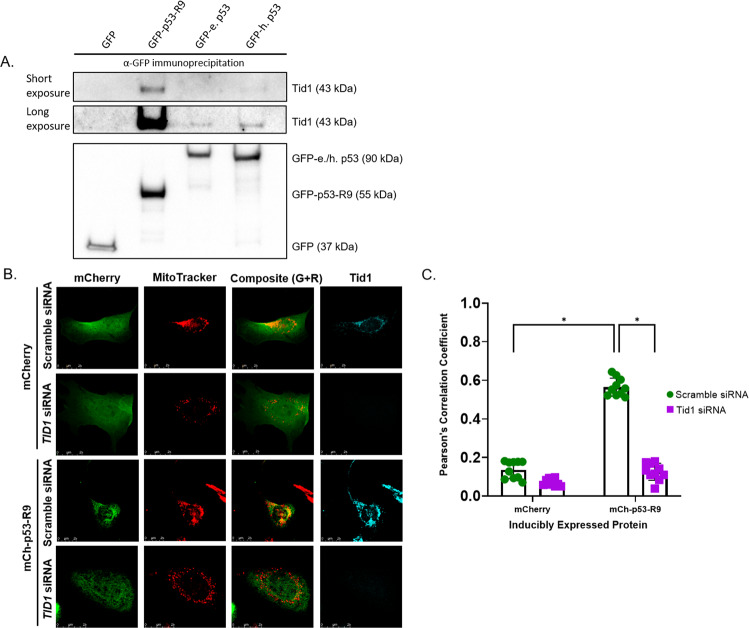
p53-R9 binds translocation chaperone Tid1, and p53-R9 localization at the mitochondria requires Tid1 expression. **A** U2OS *TP53*^−/−^ cells were transfected to express GFP, GFP-p53-R9, GFP-e. p53, or GFP-h. p53. Cells (10^6^) were lysed 8 h after transfection, then immunoprecipitation was performed with GFP-Trap beads. Immunoprecipitates were analyzed for Tid1 and GFP expression via immunoblot. **B** Representative confocal images of cells expressing mCherry or mCherry-p53-R9 and labelled with mCherry antibody (Green), MitoTracker (Red), and Tid1 antibody (Cyan) show the localization of the inducible protein (mCherry or mCh-p53-R9) in the presence or absence of transport chaperone protein Tid1 after transfection with scramble or Tid1 siRNA. Composite image includes both the green and red channels to show colocalization. Tid1 knockdown results in a punctate pattern in the MitoTracker signal, as previously published (Ng A, 2014). **C** Colocalization of mCherry antibody and MitoTracker signals determined by Pearson’s Correlation Coefficient after Otsu auto threshold applied to remove background. *N* = 100 cells from ten separate images visually positive for both mCherry and MitoTracker signal prior to background removal. There is significantly more correlation between the mCherry signal and the MitoTracker signal in mCh-p53-R9 (Scramble siRNA) vs both mCherry (Scramble siRNA) and mCh-p53-R9 (Tid1 KD); Two-Way ANOVA with Tukey’s multiple comparison test and correction *p < 0.0001. Error bars represent standard deviation of ten images collected across three experimental replicates (3 or 4 images/replicate).

The tumorous imaginal disc 1 (Tid1) protein is responsible for mitochondrial translocation of p53 in response to cellular stress; the DNAJ domain of Tid1 binds to both the N- (1–98) and C-terminus (293–393) of p53 [27, 28]. To determine if Tid1 binds to p53-R9, we transfected GFP-tagged p53 vectors into U2OS *TP53*^−/−^ cells and performed immunoprecipitation with GFP-trap beads. We identified robust binding of Tid1 to p53-R9 (Fig. 6A). Tid1 also immunoprecipitated with GFP-tagged human and elephant p53, but to a lesser extent despite similar quantity of the p53 proteins pulled-down as bait for Tid1 (Fig. 6A)

To determine if Tid1 is required for p53-R9 localization at the mitochondria, we used siRNA to knock down Tid1 in cells expressing mCherry-p53-R9 or mCherry. A scrambled version of the siRNA was included as a negative control. We imaged cells using confocal microscopy that were stained for Tid1 (aqua blue), mitochondria (red), and mCherry (green) in U2OS *TP53*^−/−^ cells expressing mCherry or mCherry-p53-R9 protein. We compared the colocalization of p53-R9 and MitoTracker in the presence and absence of Tid1. Supporting the observation shown in Fig. 5A, we observed significantly more co-localization between the mitochondria and p53-R9 than the mitochondria and the mCherry protein alone (*p* < 0.0001, Two-way ANOVA) (Fig. 6B, C). We also observed a striking similarity between the fluorescence patterns of p53-R9, the mitochondria, and Tid1 within the cell (Fig. 6B). To determine if Tid1 is required to localize p53-R9 at the mitochondria, we compared the colocalization of p53-R9 and MitoTracker in the presence and absence of Tid1 protein expression. The colocalization of p53-R9 and MitoTracker was significantly diminished in Tid1 knock down cells compared to cells treated with scramble siRNA (*p* < 0.0001, Two-way ANOVA) (Fig. 6B, C). The pattern of p53-R9 localization in the absence of Tid1 resembles that of mCherry alone; when Tid1 is knocked down, p53-R9 no longer specifically localizes to the mitochondria and p53-R9 is expressed throughout the cell. In cells with Tid1 knocked down there is more p53-R9 signal dispersed throughout the cytoplasm, compared to cells expressing Tid1, which suggests that Tid1 is required for p53-R9 to localize at the mitochondria. Similar results were confirmed with an siRNA targeting a different region of Tid1 (Fig. SI6a–d).

**Fig. 7:**
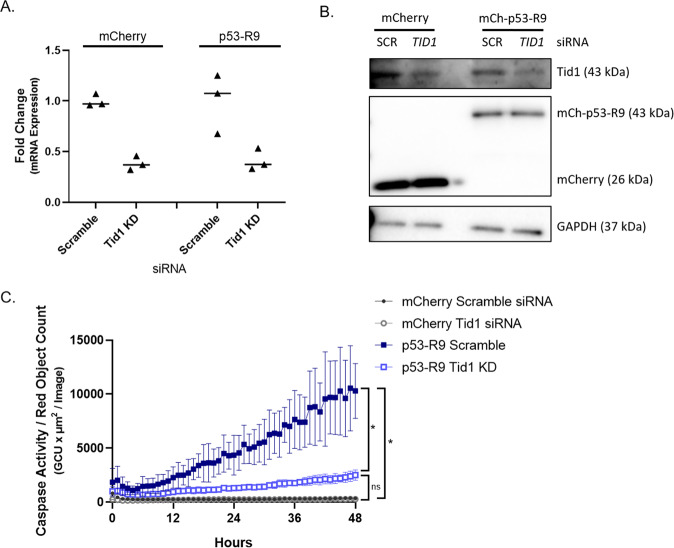
Tid1 knockdown significantly decreases the ability of p53-R9 to induce apoptosis. **A** qPCR confirming Tid1 knockdown in mCherry and mCh-p53-R9 expressing U2OS *TP53*^−/−^ cells at the mRNA level. **B** Immunoblot confirming Tid1 knockdown in mCherry and mCh-p53-R9 U2OS *TP53*^−/−^ cells at the protein level. Blots were probed with antibodies to Tid1, mCherry, and GAPDH. **C** U2OS *TP53*^*−/−*^ cells expressing mCherry or mCh-p53-R9 were transfected with scramble or Tid1 siRNA and analyzed for Caspase-3/7 activity (Caspase-3/7 green apoptosis assay reagent used with the Incucyte Live-Cell Imaging and Analysis System). Area under the curve (AUC) was calculated for *N* = 3 experiments; Welch’s *t* test of AUCs from mCherry scramble siRNA versus mCh-p53-R9 scramble siRNA and mCh-p53-R9 scramble siRNA versus mCh-p53-R9 Tid1 siRNA, *p < 0.05. Welch’s *t* test of AUCs from mCherry Tid1 siRNA versus p53-R9 Tid1 siRNA, ns *p* > 0.05. Error bars represent standard deviation of three images/well taken in three wells of a representative experiment.

We next tested the hypothesis that Tid1 is required for p53-R9 to induce apoptosis. Tid1 was knocked down in U2OS *TP53*^−/−^ cells, as measured by quantitative rt-PCR and Western blot (Fig. 7A, B) Cells expressing mCh-p53-R9 treated with scramble siRNA had significantly higher caspase activity than cells expressing mCherry treated with the same siRNA (Fig. 7C). Also, mCh-p53-R9 expressing cells treated with Tid1 siRNA had significantly less caspase activity than cells expressing mCh-p53-R9 that were treated with control siRNA (Fig. 7C). These results support the hypothesis that p53-R9 induces apoptosis at the mitochondria through a Tid1 dependent mechanism.

### p53-R9 interacts with pro-apoptotic protein Bax and cytochrome c is released from the mitochondria with p53-R9 expression

**Fig. 8:**
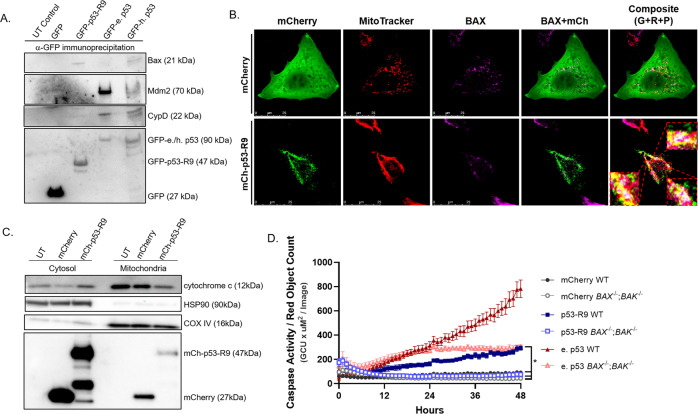
p53-R9 binds to Bax and *BAX*/*BAK* are necessary for p53-R9 induced apoptosis. **A** U2OS *TP53*^−/−^ cells were transfected to express GFP, GFP-p53-R9, GFP-elephant p53, GFP-human p53, or untransfected. 8 hours post-transfection, cell lysates (10^6^ cells) were immunoprecipitated using GFP-Trap beads to pull down GFP, GFP-p53-R9, GFP-e. p53, or GFP-h. p53. Immunoprecipitants were run on immunoblots and analyzed for presence of Bax, Mdm2, CypD, and GFP. **B** U2OS *TP53*^−/−^ cells expressing mCherry or mCh-p53-R9 were stained and analyzed for co-localization of mCh-p53-R9 and BAX at the mitochondria. Cells were stained with antibody to BAX (purple), antibody to mCherry (green), and MitoTracker (red). Representative confocal images are shown. Composite image shows green, red, and purple signal. Overlapping BAX (purple), mCh-R9 (green), and mitochondrial (red) signal are white and shown close-up in the inset images. **C** Cytochrome c is released into the cytosol with p53-R9 expression. U2OS *TP53*^−/−^ cells were transfected to express mCherry or mCh-p53-R9. Cell lysates were fractionated to separate cytosol and mitochondria. Immunoblots were performed to measure expression of cytochrome c and mCherry or mCh-p53-R9 in each fraction. Mitochondria protein HSP90 and cytosol protein COXIV were measured to confirm separation of the fractions. **D** DLD1 WT and DLD1 *BAX*^−/−^*/BAK*^−/−^ cells expressing mCherry, mCh-p53-R9, or mCh-e. p53 were analyzed for Caspase-3/7 (Caspase-3/7 green apoptosis assay reagent used with the Incucyte Live-Cell Imaging and Analysis System). Area under the curve (AUC) was calculated for *N* = 3 experiments. p53-R9 induces significantly more apoptosis than mCherry, regardless of cell genotype, and significantly more apoptosis in WT cells than *BAX*^−/−^;*BAK*^−/−^ cells; One-way ANOVA with Dunnett Correction comparing p53-R9 (WT) vs. mCherry (WT), mCherry (*BAX*^−/−^;*BAK*^−/−^), and p53-R9 (*BAX*^−/−^;*BAK*^−/−^), *p < 0.01. Error bars represent standard deviation of 3 images/well taken in three wells of a representative experiment.

To determine the mechanism by which p53-R9 expression induces apoptosis, we interrogated its ability to bind to pro- and anti-apoptotic mitochondrial, BCL-2 family members. Human p53 binds to BCL-xL, BCL-2, and Bak [13, 14]. The DNA binding domain of p53 binds to Mcl-1, but less readily than BCL-xL [12, 29]. BCL-xL binds to a C-terminally truncated, 220 amino acid long p53, but not a C-terminally truncated, 100 amino acid protein [14]. Characterization of the BCL-xL binding domain of p53 demonstrated that an intact DNA binding domain is required for this interaction [17, 30]. The DNA-binding-domain of p53 (102-292) can induce Bak oligomerization [31]. Bak binds to p53 that is C-terminally truncated at amino acid 160, but not at amino acid 93 [13]. There have been conflicting reports regarding a physical interaction between Bax and p53, potentially due to the low levels of expression of this protein that make it difficult to detect [14, 32]. It has been hypothesized that the activation of Bax by p53 occurs through a transient physical interaction [11]. We surveyed various BCL-2 family members for binding to p53-R9 by immunoprecipitation. We did not detect BCL-xL, BCL-2, or Bak after pull-down of p53-R9, elephant p53, or human p53 (Data not shown). We did immunoprecipitate Bax with p53-R9 (Fig. 8A). These results suggest that p53-R9 interacts with Bax, and this interaction may activate Bax to cause mitochondrial outer membrane permeabilization.

Additionally, MDM2 immunoprecipitated with both elephant and human p53, but no band was observed in the lane with p53-R9 (Fig. 8A), potentially due to an alteration at one of the MDM2 binding sites in p53-R9 (W23G) [2, 33]. This result contradicts previous results suggesting co-immunoprecipation of p53-R9 with MDM2 [1]. It remains possible that these two proteins interact, but that the interaction is weak as suggested recently by Padariya et al. [34]. The mitochondrial pore protein cyclophilin D, which regulates necrotic cell death, co-immunoprecipitated with both elephant and human p53 [35]. However, we did not detect co-immunoprecipitation of cyclophilin D with p53-R9 (Fig. 8A), suggesting that p53-R9 induces cell death through apoptosis and not necrosis.

To determine if p53-R9 interacts with Bax at the mitochondria, we imaged cells expressing either mCherry or mCh-p53-R9. Cells were stained with MitoTracker (red), mCherry antibody (green), and Bax antibody (purple). Confocal imaging revealed signal overlap between mCh-p53-R9, Bax, and mitochondria (Fig. 8B signal overlap in composite image is white), further supporting that p53-R9 induces apoptosis after transport to the mitochondria through interaction with Bax.

To strengthen our hypothesis that p53-R9 induces apoptosis at the mitochondria, we fractionated cells to separate the cytosol and mitochondria. Western blots on the separated fractions confirmed that p53-R9 is present in the mitochondria. We further observed that cytochrome c is released into the cytosol in cells expressing p53-R9, but not in control cells (Fig. 8C). These results strongly suggest that p53-R9 induces mitochondrial-mediated apoptosis.

Binding to Bax can potentially explain how p53-R9, after being transported to the mitochondria by Tid1, induces caspase activation, cytochrome c release, and apoptosis. To determine if Bax and another pro-apoptotic BCL-2 family member, Bak, are necessary for p53-induced apoptosis, we transfected a *BAX*^−/−^;*BAK*^−/−^ colorectal adenocarcinoma cell line (DLD-1) and the parental cell line that expressed wild type *BAX* and *BAK* with p53-R9. Despite not observing an interaction between p53-R9 and Bak, we chose the double knock-out cell line because we were also unable to pull-down Bak with its known binding partner human p53 and therefore could not rule out p53-R9/Bak binding [14]. p53-R9 induced apoptosis in wild-type DLD-1 cells but did not induce apoptosis in *BAX*^−/−^;*BAK*^−/−^ DLD-1 cells (Fig. 8D). This result indicates that either Bax, Bak, or both proteins are necessary for p53-R9 to induce apoptosis in human cancer cells.

## Discussion

The ability of tumor suppressor p53 to induce apoptosis in the absence of its role as a transcription factor has been studied for a quarter-century [36–38]. Its ability to induce transcription-independent apoptosis has been attributed to its functions at the mitochondria, the central death regulator of the cell [14]. Targeting p53, or monomeric p53 subdomains, to the mitochondria is documented to be sufficient to induce apoptosis in various cancer cell lines [39–41]. Here we show that p53-R9, a naturally-occurring truncated elephant p53, binds to Tid1 which then traffics it to the mitochondria. This mitochondrial localization of p53-R9 induces apoptosis in human cancer cells by interacting with Bax, inducing cytochrome c release, and subsequently activating caspases, despite the truncated protein’s inability to regulate key p53 target genes. If p53-R9 functions the same way in elephant cells, then p53-R9 could work either additively or synergistically with full-length elephant p53 to cause transcription-independent apoptosis, in addition to the transcription-dependent apoptosis induced by ancestral elephant p53. Defining the function of elephant *TP53* retrogenes in human cancer cells can direct future studies to characterize its role in elephant cells, which will help us better understand how elephants overcome the inherent oncogenic risk of body size and longevity. Furthermore, our efforts to document p53-R9 function in human cells may inform translational efforts to develop therapeutics for human cancers.

In the current study, we focused on p53-R9’s ability to induce apoptosis in a human cancer cell line that does not express endogenous human p53. We acknowledge a weakness of this investigation may be that p53-R9 functions differently or through additional mechanisms in the presence of full-length ancestral elephant p53. Further studies are now required to determine if p53-R9 has additional p53-dependent functions. One question to answer beyond the scope of our study is whether p53-R9 can affect transactivation of p53 target genes when ancestral elephant p53 is present. Another potential shortcoming of the current work is that the experiments were performed in human cancer cells not elephant cells due to lack of molecular biological tools to interrogate elephant-specific genes. Additional studies will need to determine if p53-R9 induces apoptosis at the mitochondria in elephant cells, if elephant-focused tools become available. The generation of elephant-specific tools and reagents will greatly aid future studies focused on defining the mechanisms of action of p53-R9 in elephant cells.

In addition to *TP53* copy number expansion in their genomes, other potential mechanisms of tumor defense have been documented in elephants. For example, elephants have extra copies of *LIF*, one of which induces apoptosis when expressed in response to DNA damage [42]. These two genes, *LIF6* and *TP53-R9* are transcribed in elephant tissue and each function to induce apoptosis [2, 42]. Other cancer-related genes and biological processes are enriched in accelerated genomic regions of elephants including FANCL and TNF-mediated signaling pathway [3, 43]. These other potential mechanisms of cancer resistance need to be functionally investigated and can be explored in the future for possible interactions with p53 retrogenes.

Our findings point to a potential explanation for how elephants defend against the oncogenic risk of many cells dividing over a long period of time. If p53-R9 induces transcription-independent apoptosis at the mitochondria in elephant cells, then this additional apoptotic mechanism may help explain the lower threshold for DNA-damage-induced apoptosis in elephant cells compared to human cells. Understanding the mechanism by which a naturally occurring, truncated elephant p53 protein induces apoptosis, in the absence of a full-length p53, lays the groundwork to harness the evolutionary advantage of cancer resistance in elephants to treat human cancers. Elephant p53 retrogenes could play an adjunct role in future cancer therapies and lead to increased cancer cell death for human patients.

## Methods

### Cell lines

Female U2OS (ATCC) were grown in McCoy’s 5A Medium (Gibco) with 10% fetal bovine serum (FBS). Female SAOS-2, *TP53*^−/−^, (ATCC) were grown in and grown in McCoy’s 5A Medium (Gibco) with 15% FBS. Male T98G, mut*TP53*^M273I^, (ATCC) were grown in Eagle’s Minimum Essential Medium (Gibco) with 10% fetal bovine serum (FBS). Male HCT116 WT and HCT116 *TP53*^−/−^ (Horizon) were grown in McCoy’s 5A Medium (Gibco) with 10% FBS. Male DLD1 WT and DLD1 *BAX*/*BAK* (Sigma-Aldrich) were grown in RPMI Medium 1640 (Gibco) with 10% FBS. All media was supplemented with 5% GlutaMAX (Gibco), 5% Pen Strep (Gibco), 5% HEPES (Gibco) and 5% Sodium Pyruvate (Gibco). The cell lines were not used at passage numbers higher than 20. Cell lines were tested regularly for mycoplasma. All cells were grown at 37 *°*C.

### Caspase 3/7 and annexin V cell death assays

We used the Caspase-Glo 3/7 Assay system (Promega G8090) to quantify the activation of caspases after transfection and sorting for initial apoptosis screen in cell lines SAOS-2, T98G, HCT116, and U2OS. Caspase values were normalized viability as measured by CellTiter-Glo Luminescent Cell Viability Assay (Promega G7570). Further apoptosis assays utilized the Incucyte Live-Cell Imaging System. Cells were transfected, and 10,000 cells were sorted for a fluorescent marker into 3–6 wells of a 96-well plate (100 μL of growth media). To detect apoptosis, Caspase-3/7 Green reagent (Essen) was added 1:1000 in each well. To detect cell death, Annexin V Green reagent (Essen) was added 1:100 in each well. Cells were imaged every 2 h for 48 h and images were analyzed with Incucyte Software. In Caspase-3/7 assays, green intensity/image was normalized to red object count/image. In Annexin V assays, green object count/image was normalized to red object count/image. Cells expressing red fluorescent proteins were added to wells containing 50 µM Z-VAD-FMK, when noted. In assays validating Z-VAD-FMK, Annexin V green object count was normalized to cell confluence.

### Vectors

Backbones for vectors encoding fluorescently tag proteins were pcDNA3.1-mCherry and pcDNA3.1-GFP. The stop codon was removed from the tag and *TP53-R9*, elephant *TP53*, truncated elephant *TP53*, human *TP53*, or truncated human *TP53* with the start codons removed were added directly after the tag. To generate stable cell lines, we used the pLVX-TetOne-Puro Vector-mCherry and the same backbone with *TP53-R9* with the start codon removed inserted immediately following the mCherry tag with the stop codon removed. The vectors encoding for elephant proteins were codon optimized for expression in human cells (Genscript). The cell surface localization vector was generated by inserting the partial coding sequence for CD4 (UniProt Q13969) at the N-terminus prior to the start codon of the pcDNA3.1-mCherry-p53-R9 vector (Genscript).

### CRISPR/CAS9 *TP53* knockout

U2OS cells were transfected with p53 CRISPR/Cas9 KO Plasmids (Santa Cruz Biotechnology) with Lipofectamine 2000 (Thermo Fisher). After 2 days, cells were treated with 10 µM Nutlin-3 for 14 days to enrich for *TP53*^−/−^ cells. Following Nutlin-3 treatment, monoclonal cell lines were generated by limiting dilution. Cell lines were grown and screened for *TP53* status via immunoblot; then Sanger sequencing was used to identify specific mutation in the *TP53* gene.

### Tid1 knockdown

Cells were treated with DNAJA3 Silencer Select siRNA (Invitrogen #137909 used in Figs. 6, 7 and #137907 used in Fig. SI6) or Silencer Select Negative Control #1 siRNA (Invitrogen #4390844) in Lipofectamine RNAiMAX (Invitrogen) for 24 h, then cells were harvested for and further experiments were carried out over the next 5 days.

### Immunoblotting

Cells were lysed with Cell Lysis Buffer (Cell Signaling Technology) supplemented with Protease/Phosphatase Inhibitor Cocktail (Cell Signaling Technology). For Western blot to confirm expression of membrane-localized CD4 fusion protein in Fig. 5B, cells were lysed in RIPA buffer made using recipe found here: https://www.abcam.com/protocols/general-western-blot-protocol#Solutions%20and%20reagents. Lysates were mixed with NuPAGE LDS Sample Buffer (Life Technologies) and NuPAGE Sample Reducing Agent (Life Technologies) and were run on Bolt 4–12% Bis-Tris Plus gels (Invitrogen). Gels were transferred with iBlot2 PVDF Mini Stacks (Invitrogen) and iBlot2 (Invitrogen). Blots were probed with antibodies for mCherry (GTX128508, GeneTex), GFP (sc-99G, Santa Cruz Biotechnology), Tid1 (EPR12414, abcam or RS-11, Santa Cruz Biotechnology), p53 (DO-1, Santa Cruz Biotechnology), CHOP (9C8, Novus Biologicals), ATF6-N (70B1413.1, Novus Biologicals), pEIF2a (9721, Cell Signaling Technology), EIF2a (9722, Cell Signaling Technology), Bax (6A7, Santa Cruz Biotechnology), MDM2 (sc-965, Santa Cruz Biotechnology), Cyclophillin D (E11AE12BD4, abcam), Hsp90 (C45G5, Cell Signaling Technology), Cox IV (3E11, Cell Signaling Technology), and GAPDH (D4C6R, Cell Signaling Technology). Blots were imaged using Bio-Rad ChemiDoc MP Imaging System or Azure Biosystems Sapphire Biomolecular Imager.

### Transfection and sorting for fluorescence

Cells were transfected with either Lipofectamine 3000 (Invitrogen) (T98G), or electroporated with either the Nucleofector (Lonza) (SAOS-2) or with the Neon (Invitrogen) (HCT116 WT, HCT116 *TP53*^−/−^, U2OS WT and U2OS *TP53*^*−/*−^). Cells were sorted through the University of Utah Cores Facility using the BD FACSAria II, BD Fortessa, Propel Labs Avalon, or they were sorted using the Sony SH800S. On each machine, the cells were sorted to exclude dead cells, exclude doublets, then cells were sorted for red or green fluorescence into a 96-well plate.

### Luciferase and qPCR gene expression assays

For Nano-Glo Dual-Luciferase Reporter Assay system (Promega), cells were triply transfected with a luciferase vector, Nano-GloDLR control vector and a vector encoding our gene of interest, plated into a 96 well plate and luminescence was measured using SpectraMax iD5 (Molecular Devices). PG13-luc (wt p53 binding sites) was a gift from Bert Vogelstein (Addgene plasmid # 16442; http://n2t.net/addgene:16442; RRID:Addgene_16442). pGL4.38 [*luc2p*/p53 RE/Hygro] containing two copies of a p53 response element was purchased from Promega. Two-step qPCR was performed using cDNA made RT^2^ First Strand Kit (Qiagen) using RNA extracted from cells using RNeasy Plus Mini Kit. Probes for MDM2 (Hs01066938_m1), GADD45a (Hs00169255_m1), CDKN1A (Hs00355782_m1), BAX (Hs00180269_m1), APAF1 (Hs00559441_m1), PIDD1 (Hs00611142_g1), GAPDH (4310884E), and DNAJA3 (Hs00170600_m1) were purchased from Thermo Fisher. Express qPCR Supermix (Invitrogen) reactions were loaded onto MicroAmp Fast Optical 96-well reaction plates (Applied Biosystems) and read on StepOnePlus Real-time PCR System (Applied Biosystems). TaqMan Gene Expression probes were used to amplify all target genes (Applied Biosystems). To normalize between conditions, *C*_t_ values of target genes were normalized to *C*_t_ values of GAPDH. We used these normalized values to generate log2 fold changes.

### Immunocytochemistry staining and imaging

Sorted cells were seeded onto coverslips, fixed with 4% paraformaldehyde (FB002; Invitrogen), and stained in routine manner. We stained using DAPI, MitoTracker Deep Red FM (0.1 µL/mL, M22426; Invitrogen), rabbit anti-mCherry (1:100, GTX128508; GeneTex), mouse anti-TID1_L/S_ (1:50, sc-18820; Santa Cruz Biotechnology), and anti-BAX-6A7 (1:50, sc-23959; Santa Cruz Biotechnology) with AlexaFluor 405-conjugated anti-mouse, and AlexaFluor 555-conjugated anti-rabbit secondary antibodies. Each 3D image is comprised of a Z-Stack series captured on a Leica SP8 Confocal at 63× magnification and generated utilizing Leica’s LAS X Life Science Microscope Software Platform’s 3D Viewer.

### Colocalization quantification

For all slides, we took whole-coverslip immunofluorescence images, then imaged ten representative sections of each condition containing ten cells that stained positively for our cell markers using confocal microscopy (Leica SP8). Ten images were collected from three experimental replicates. Each image was analyzed using NIS-Elements AR Analysis, background was subtracted based on Auto Thresholds which were determined using Original Otsu method, then Colocalization Analysis Plug-in was used to determine Pearson’s Correlation Coefficient for each image. PCC is a commonly used metric for measuring colocalization between two fluorophores in an image, determining the covariance of fluorophore signal intensity in each pixel [44, 45]. A supporting experiment was performed with an siRNA targeting a different region of Tid1 with similar results shown in the supplement (Fig. SI6).

### Immunoprecipitation

Cells expressing GFP or GFP-tagged proteins were harvested and resuspended in lysis buffer (10 mM Tris/Cl pH 7.5; 150 mM NaCl; 0.5 mM EDTA; 0.5% NP-40). Lysates were incubated and mixed end-over-end with GFP-Trap_A beads (Chromotek) overnight at 4 *°*C. Beads were washed in wash buffer (10 mM Tris/Cl pH 7.5; 150 mM NaCl; 0.5 mM EDTA) then resuspended and boiled in 2X SDS-sample buffer before immunoblotting.

### Cellular fractionation assays

U2OS *TP53*^−/−^ cells were transfected with pcDNA3.1-mCherry or mCherry-p53-Retro9 constructs. Cells were harvested after 48 h and processed using the Cytochrome C Release assay from Abcam (ab65311). The cytosolic and mitochondrial fractions were quantified by Bradford protein assay, then resuspended and boiled in 2× SDS-sample buffer before immunoblotting.

### Quantification and statistical analysis

One-way ANOVA with Dunnett’s correction was used to analyze the results of apoptosis and Annexin V assays when more than one comparison was being made to the negative control condition. When only one comparison was being made to the control condition or multiple experimental conditions were compared, Welch’s *t* test was used. For Incucyte assays, *N* represents the area under the curve for three separate experiments. To quantify colocalization, a Pearson’s Correlation Coefficient was generated from *N* = 10 separate images collected across three experimental replicates (3 or 4 images/replicate) each containing ten cells. To compare PCC values, a two-way ANOVA with Tukey’s Correction for multiple comparisons was used. To compare variances between any groups that were compared in t-tests or One-way ANOVAs, an F test or Brown-Forsythe test was used, respectively. There are no significant differences in variance between any of these compared groups. All statistical analyzes were conducted using Prism software. The statistical tests and parameters used in each experiment can be found in Fig. legends.

## Supplementary information

Supplemental Figures

Video 1

Video 2

Video 3

Video 4

Video 5

Video 6

Video 7

Uncropped Western Blots

Authorship change agreement

## Data Availability

Data sharing is not applicable to this article as no datasets were generated or analyzed during the current study.
