# Supplementary material for: Elephant TP53-RETROGENE 9 induces transcription-independent apoptosis at the mitochondria
Source: Cell Death Discov. 2023 Feb 16;9:66. doi: 10.1038/s41420-023-01348-7 (PMC9935553; doi:10.1038/s41420-023-01348-7)
Supplement: Supplementary file 1 — Supplemental Figures [file 41420_2023_1348_MOESM1_ESM.pdf]

Multimedia files in Figure 5

Movie 1: Live cell video of mCherry-tagged p53-R9 protein from the Incucyte Live Cell Imaging System.

Movie 2: Live cell video Mitotracker green from the Incucyte Live Cell Imaging System.

Movie 3: Live cell video composite of Movies 1 and 2 from the Incucyte Live Cell Imaging System showing co-occurrence of the two signals.

Movie 4: Video of Z-Stack series generated from confocal images of cell expressing mCherry (Green) with mitotracker red.

Movie 5: Videos of Z-Stack series generated from confocal images of cell expressing mCherry-tagged p53-R9 protein (Green) with mitotracker red.

Movie 6: Video of Z-Stack series generated from confocal images of a field of view of cells expressing GFP-tagged p53-R9 protein (green), with mitotracker (red), and nuclear stain (blue).

Movie 7: Video of Z-Stack series generated from confocal images of a field of view of cells expressing GFP-tagged CD4-p53-R9 protein (green), with mitotracker (red), and nuclear stain (blue).

Figure SI1.

a.

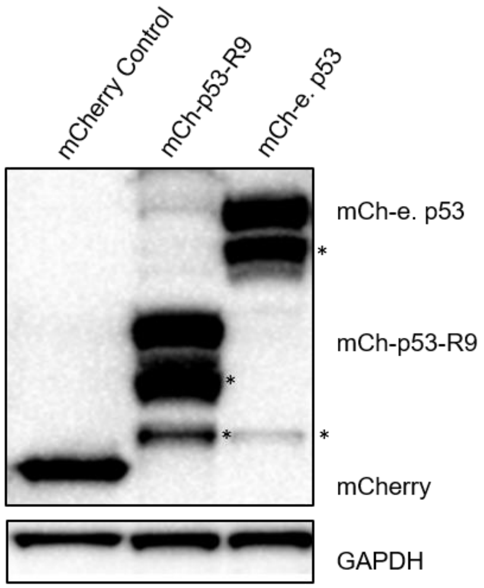

b.

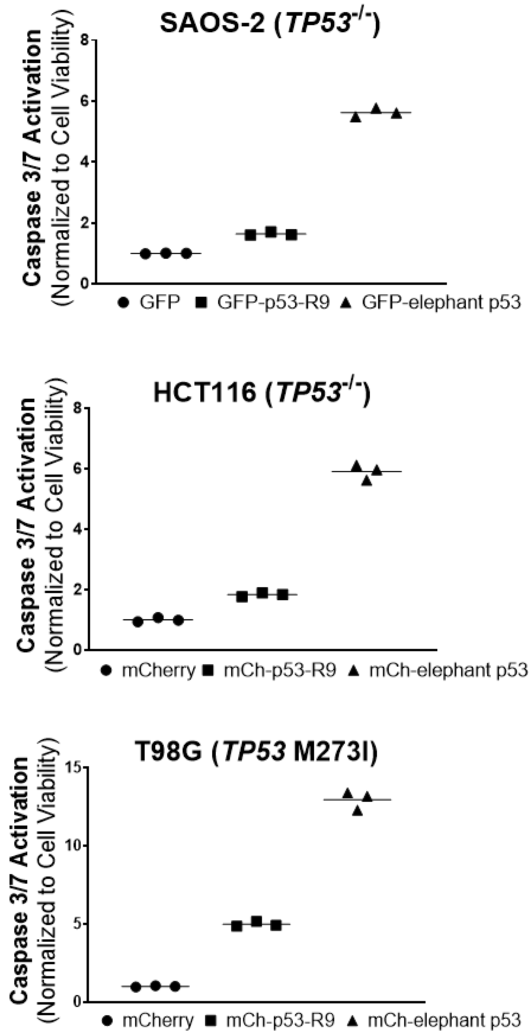

Figure SI1. Expression of p53-R9 and elephant p53 induce apoptosis of human cancer cells with mutant or null *TP53*. (a.) Western blot shows protein expression in HCT116 cells transfected to express mCherry (mCh), mCherry-p53-R9 (mCh-p53-R9), or mCherry-elephant p53 (mCh-e. p53). Protein expression was detected by probing with an mCherry antibody. Likely cleavage products are indicated by *. (b.) Transfected human cancer cells were transfected and sorted for expression of the fluorescent proteins GFP or mCherry, then analyzed for Caspase 3/7 activity using the Promega Caspase-Glo 3/7 assay, values were normalized to cell viability (Cell Titer Glo).

Figure SI2.

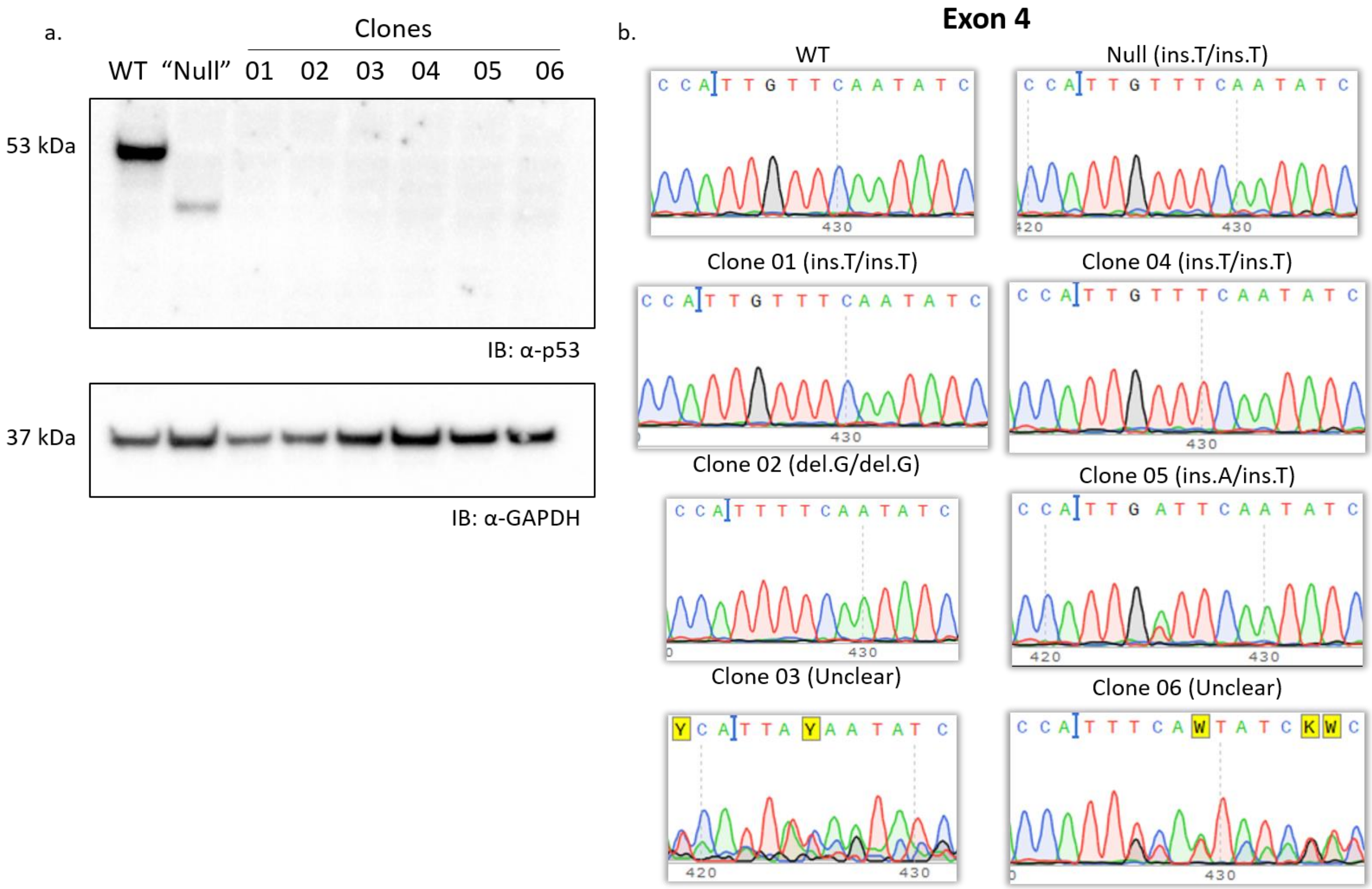
a.
Clones
WT
"Null"
01
02
03
04
05
06
53 kDa
IB: α-p53
37 kDa
IB: α-GAPDH
b.
Exon 4
WT
Null (ins.T/ins.T)
Clone 01 (ins.T/ins.T)
Clone 04 (ins.T/ins.T)
Clone 02 (del.G/del.G)
Clone 05 (ins.A/ins.T)
Clone 03 (Unclear)
Clone 06 (Unclear)

Figure SI2. Confirmation of *TP53* knockout in U2OS CRISPR edited cells (a) p53 protein expression (antibody clone: DO-1) in the parental wild type (WT) U2OS line, a previously generated *TP53* "Null" U2OS cell line, and six CRISPR edited cell lines cloned from single cells (Clones 01 – 06). (b) Sanger sequencing results confirming mutations in the six clones. Clone 5 was selected for use in this study.

Figure SI3

a.

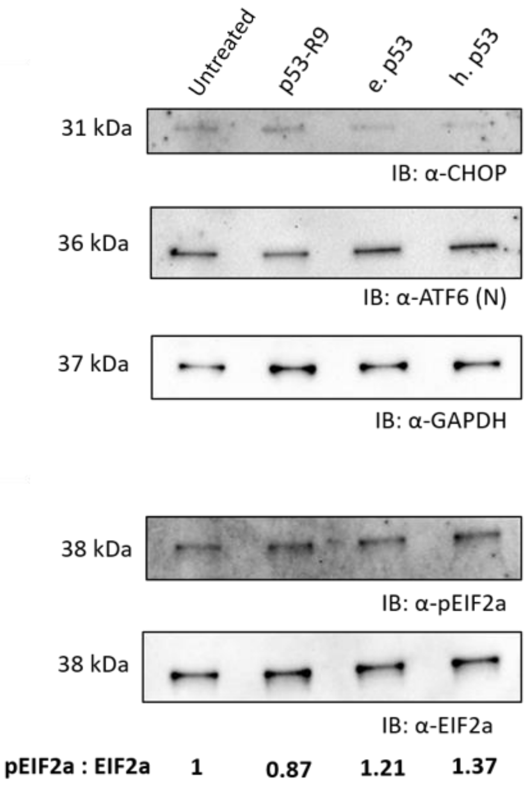

b.

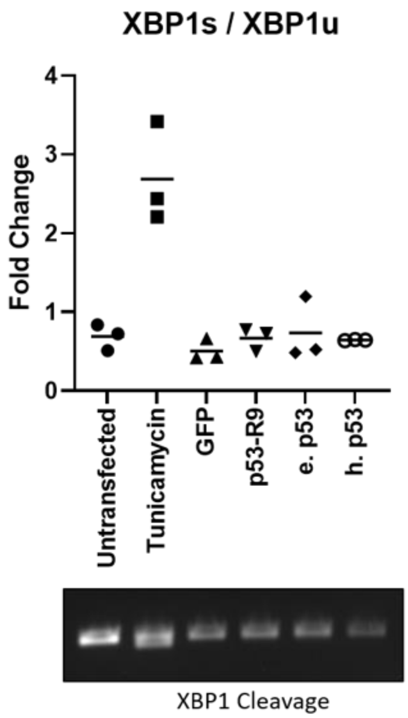

Figure SI3. Apoptosis with p53-R9 expression is not due unfolded protein response. (a) p53-R9 does not increase expression of markers of unfolded protein response. Protein expression of CHOP, ATF6 (N), GAPDH, phosphorylated EIF2a (pEIF2), and EIF2a were measured by Western blot. Values listed below are the ratio of pEIF2a and EIF2a levels. CHOP, ATF6 (N), and phosphorylation of EIF2a do not increase in response to p53-R9 expression in the cells. (b) p53-R9 expression does not induce XBP1 mRNA cleavage, which is another marker of unfolded protein response. XBP1 cleavage was quantified by qPCR and PCR followed by gel electrophoresis. qPCR probes for were designed to measure the ratio of cleaved XBP1 mRNA (XBP1s) to unprocessed XBP1 mRNA (XBP1u). The XBP1u probe anneals to the cleaved sequence, whereas the XBP1s probe anneals to sequence that spans the cleavage site. Tunicamycin (an antibiotic) was used as a positive control for ER stress and unfolded protein response.

Figure SI4.

a.

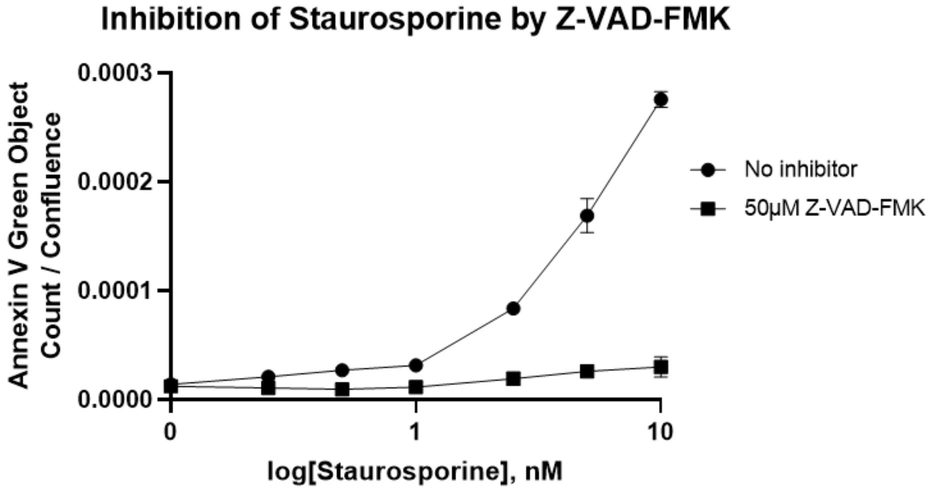

b.

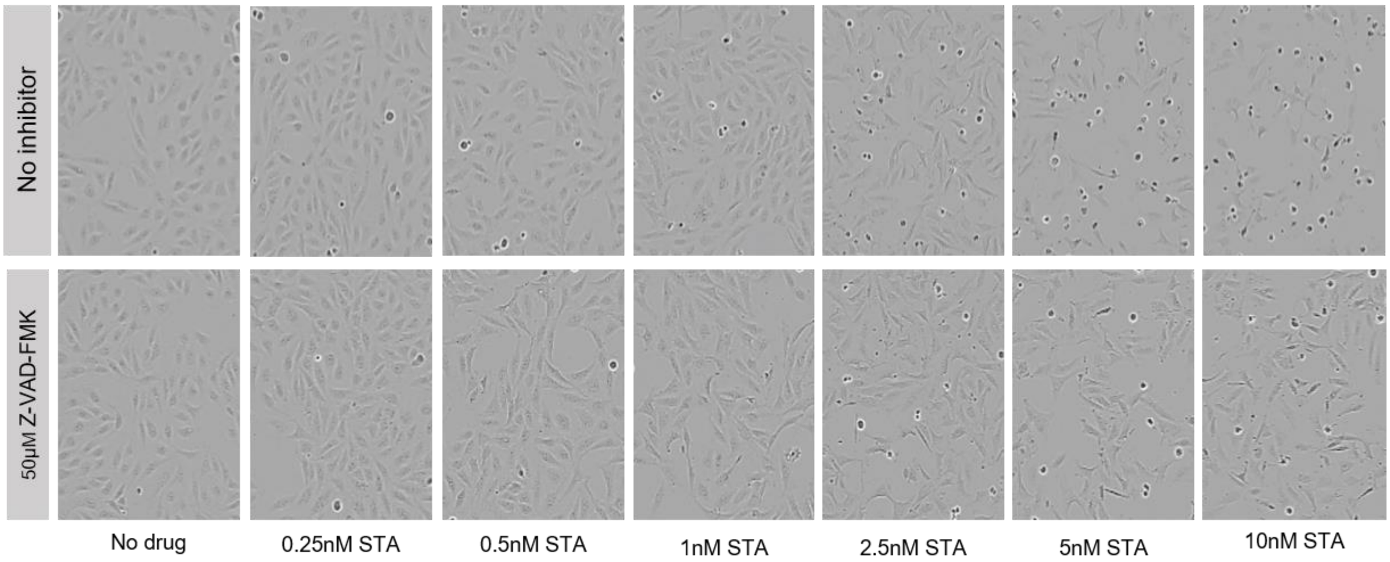

Figure SI4. Z-VAD-FMK inhibits apoptosis in U2OS *TP53*-/- cells. (a) U2OS *TP53*$^{-/-}$ cells were treated with increasing doses of Staurosporine (STA) in the presence and absence of Z-VAD-FMK and were analyzed for apoptosis (Annexin V green positive cells normalized to cell confluence) using the Incucyte Live-Cell Imaging and Analysis System. Error bars represent standard deviation. (b) Representative images of cells responding to STA in the presence and absence of Z-VAD-FMK, 48h after treatment

Figure SI5.

a.

b.

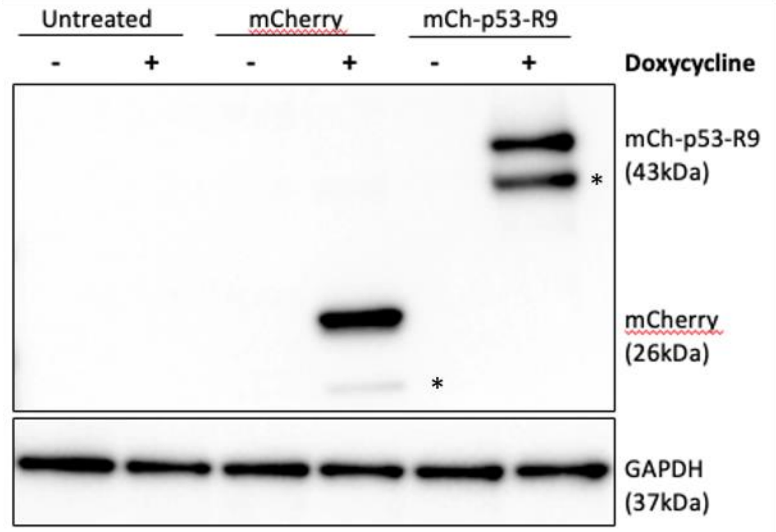

b.

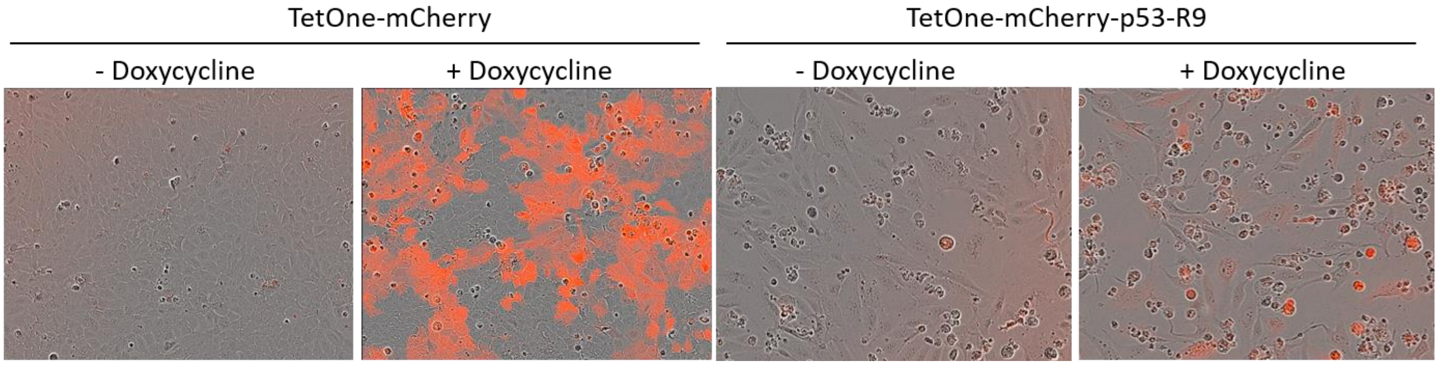

Figure SI5. Stably transduced U2OS *TP53*-/- cells express mCherry and mCherry-p53-R9 (mCh-p53-R9) with doxycycline induction. (a) Western blot was probed with mCherry antibody to measure mCherry and mCh-p53-R9 expression in U2OS *TP53*-/- cells transduced with inducible TetOne-mCherry or inducible TetOne-mCherry p53-R9 and treated or not with doxycycline. Likely cleavage products or non-specific bands are indicated by *. (b) Live cell images of red fluorescence (mCherry and mCh-p53-R9 expression) in inducible cell lines, +/- doxycycline, 24h after induction (Incucyte Live Cell Imaging System). Red signal intensity in mCh-p53-R9 cells is lower than in mCherry cells due to apoptosis induction by R9 expression. The brightest R9 expression cells are dead at this timepoint.

Figure SI6.

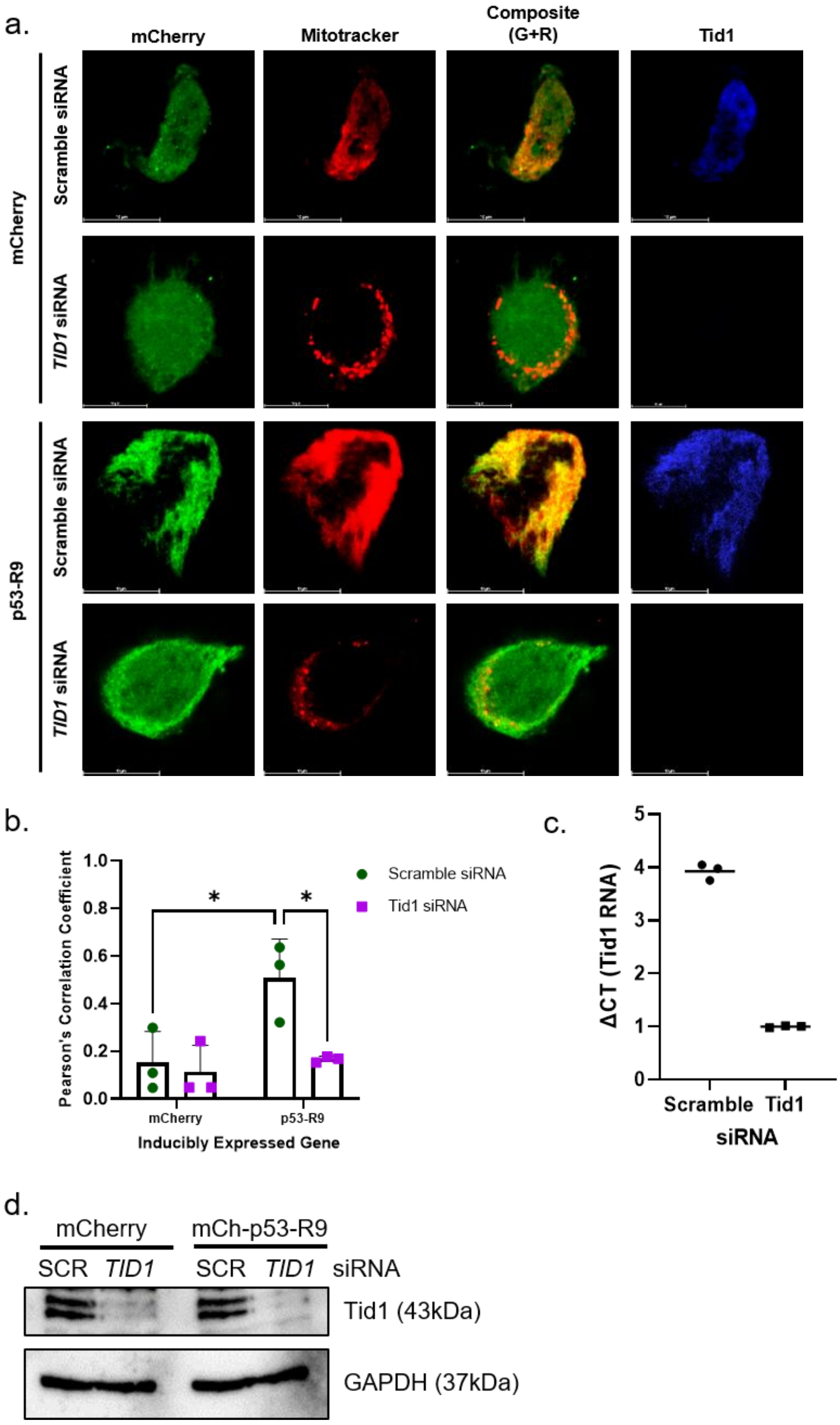
a.
mCherry
Mitotracker
Composite (G+R)
Tid1
mCherry
Scramble siRNA
TID1 siRNA
p53-R9
Scramble siRNA
TID1 siRNA
b.
Pearson's Correlation Coefficient
1.0
0.8
0.6
0.4
0.2
0.0
*
*
Scramble siRNA
Tid1 siRNA
mCherry
p53-R9
Inducibly Expressed Gene
c.
ΔCT (Tid1 RNA)
5
4
3
2
1
0
Scramble
Tid1
siRNA
d.
mCherry
mCh-p53-R9
SCR
TID1
SCR
TID1
siRNA
Tid1 (43kDa)
GAPDH (37kDa)

Figure SI6. p53-R9 localizes at the mitochondria, which is dependent upon the presence of translocation chaperone Tid1. *TID1* knockdown with an siRNA that targets a different region of *TID*1 than the one used in figure 6 confirms results. (a) Representative confocal images of cells expressing mCherry or mCherry-p53-R9 and labelled with anti-mCherry antibody (Green), Mitotracker (Red), and anti-Tid1 antibody (blue) show the localization of the inducible protein in the presence or absence of transport chaperone protein Tid1. Composite image includes both the green and red channels. (b) Colocalization of mCherry antibody and Mitotracker signals determined by Pearson's Correlation Coefficient after Otsu auto threshold applied to remove background. *N*=30 cells from 3 separate images visually positive for both mCherry and mitotracker signal prior to background removal. Two-Way ANOVA with Tukey's correction * $p< 0.05$. Error bars represent SD of 3 images/well taken in 3 wells. (c) qPCR to confirm knockdown of Tid1 at the mRNA level. (d) Immunoblot confirming Tid1 protein knockdown in the two cell lines at the time of fixation for imaging.

Figure SI7.

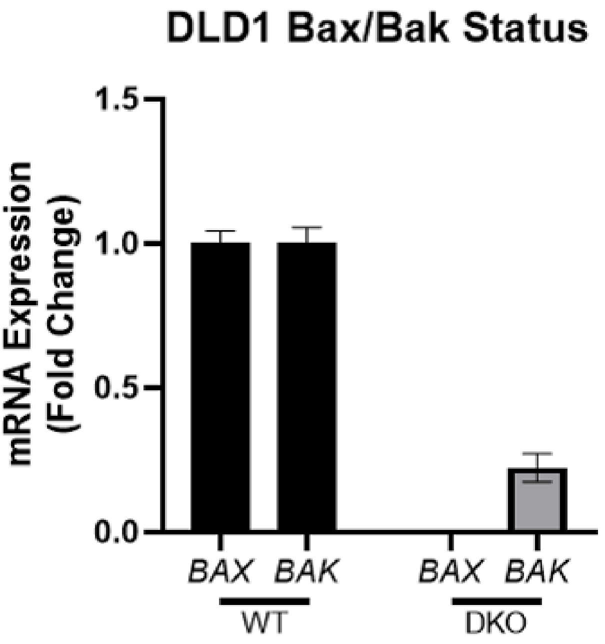
DLD1 Bax/Bak Status
mRNA Expression
(Fold Change)
1.5
1.0
0.5
0.0
BAX
BAK
BAX
BAK
WT
DKO

Figure SI7. DLD *BAX*/*BAK* knockout cells do not express *BAX* and have reduced expression of *BAK*. Quantitative rt-PCR of *BAX* and *BAK* mRNA expression in DLD1 WT and DLD1 $BAX^{-/-}$/$BAK^{-/-}$ cells, normalized to expression in DLD1 WT cells. Error bars represent standard deviation of 3 replicate wells.
