# Supplementary material for: Elephant TP53-RETROGENE 9 induces transcription-independent apoptosis at the mitochondria
Source: Cell Death Discov. 2023 Feb 16;9:66. doi: 10.1038/s41420-023-01348-7 (PMC9935553; doi:10.1038/s41420-023-01348-7)
Supplement: Supplementary file 9 — Uncropped Western Blots [file 41420_2023_1348_MOESM9_ESM.pdf]

Blots from 2A

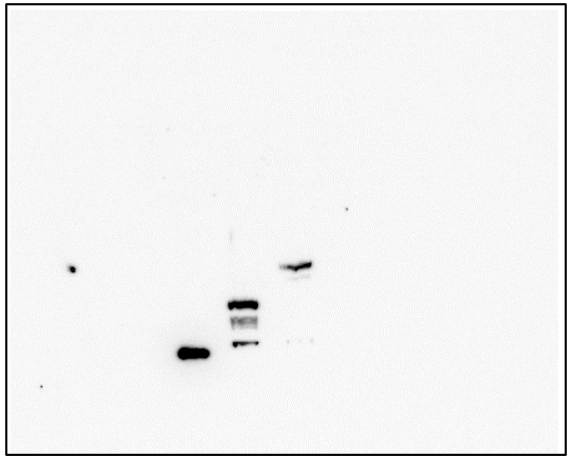

GFP

Blots from 3A

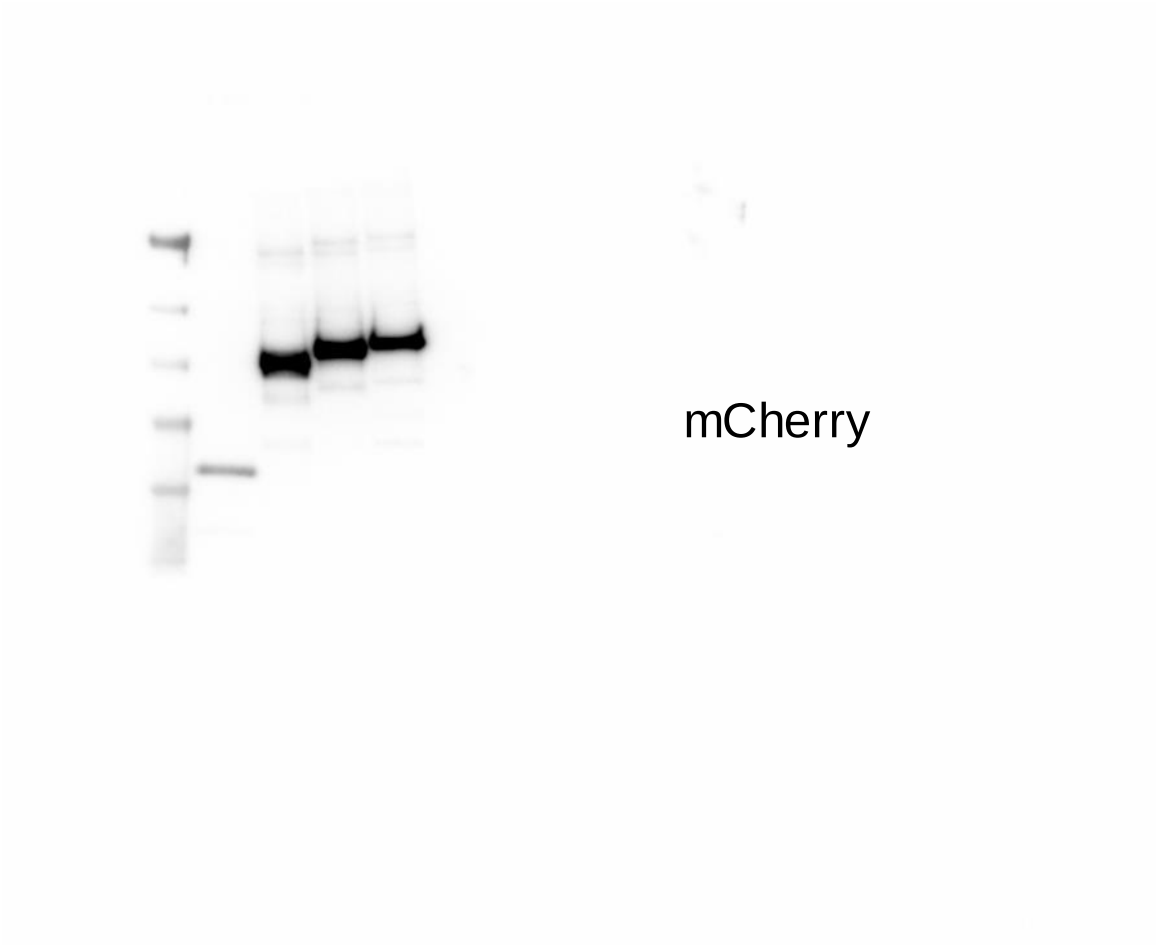

mCherry

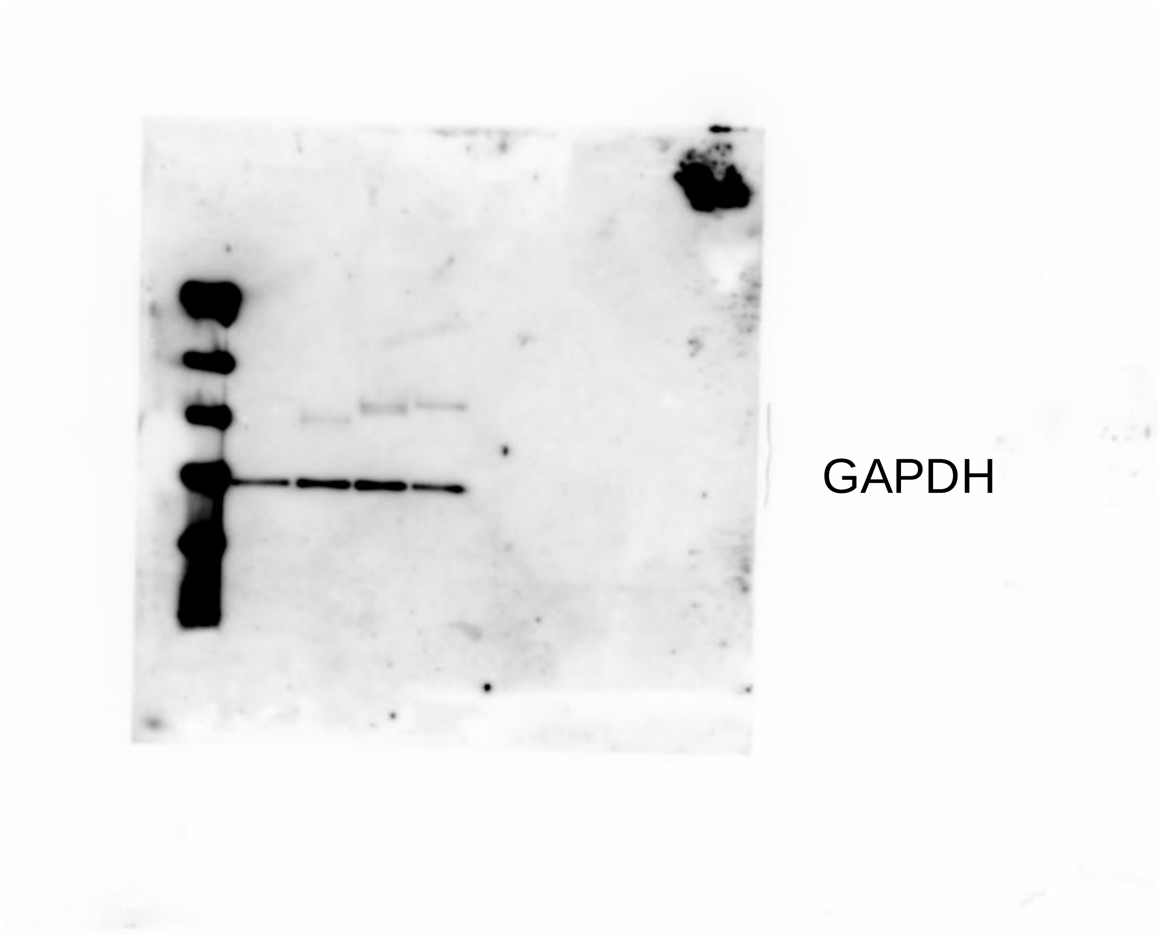

GAPDH

Blots from Figure 5B.

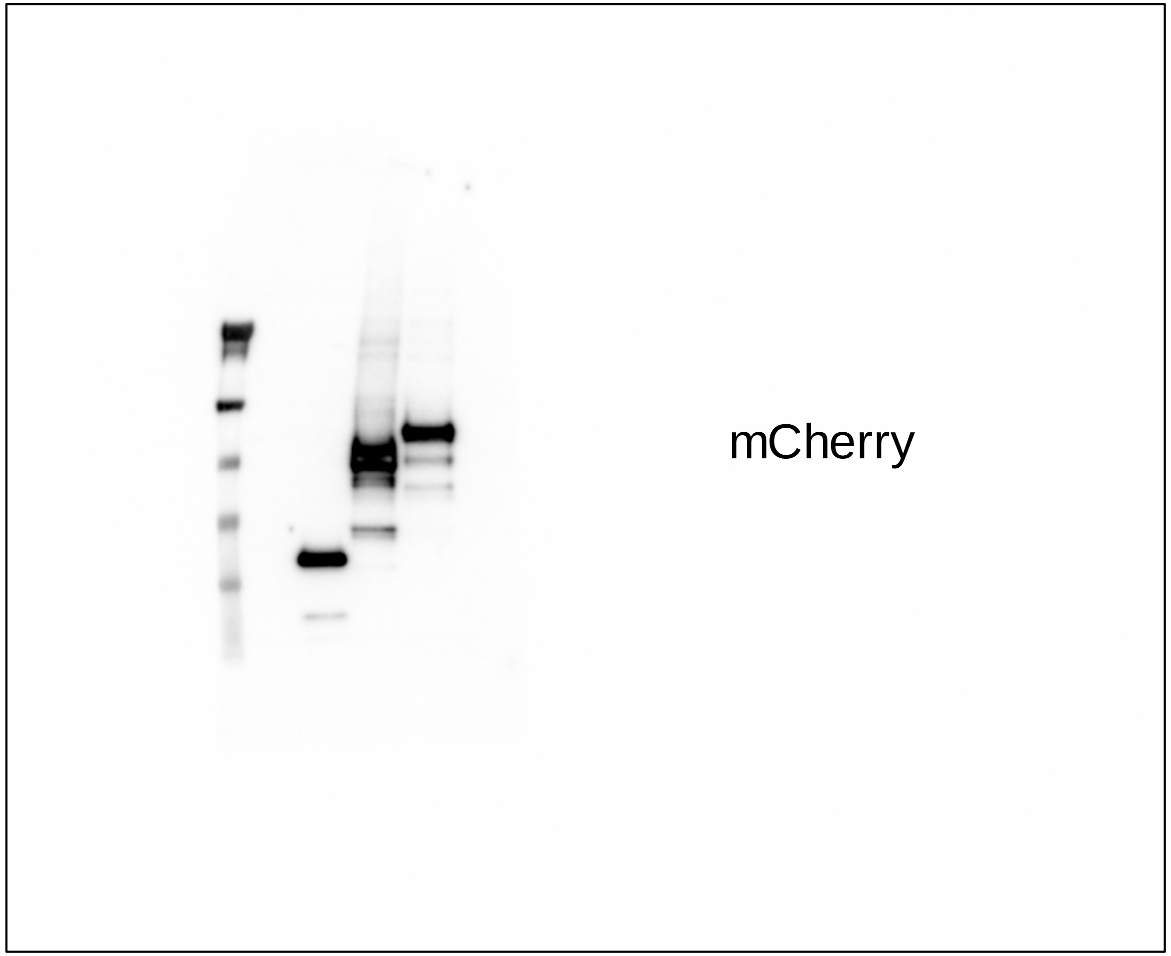

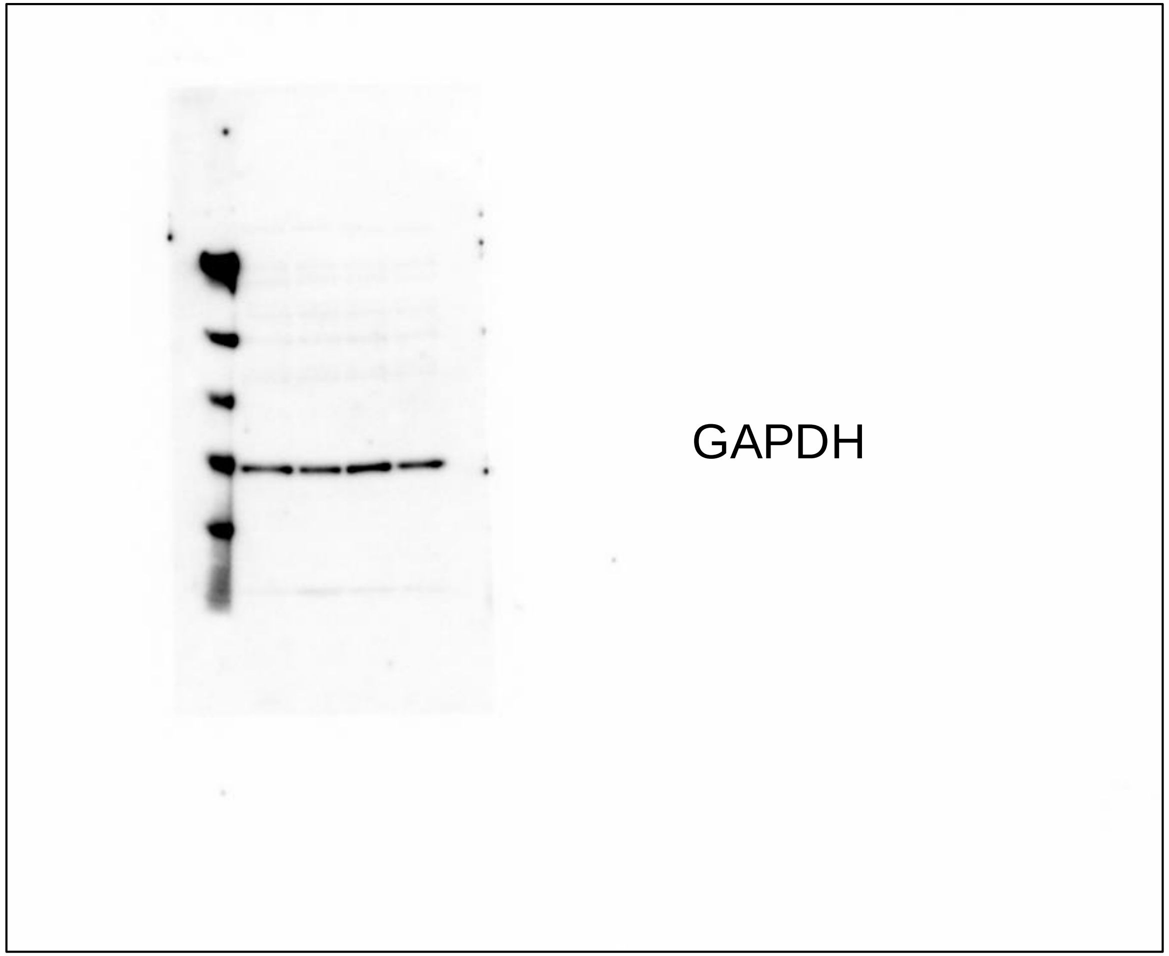

Blots from 6A.

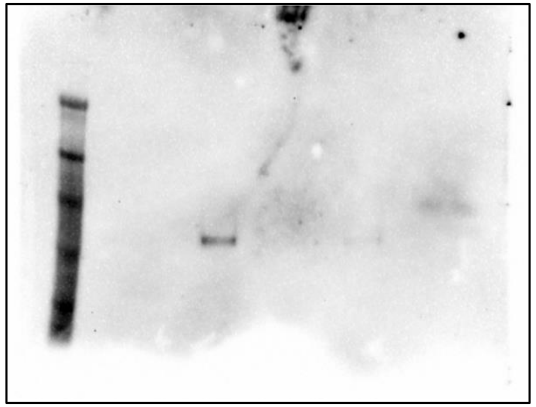

Tid1 short exposure

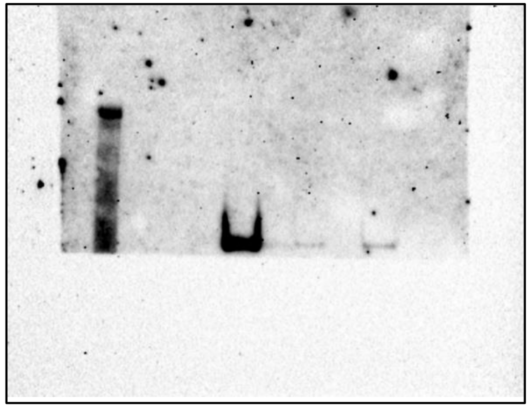

Tid1 long exposure

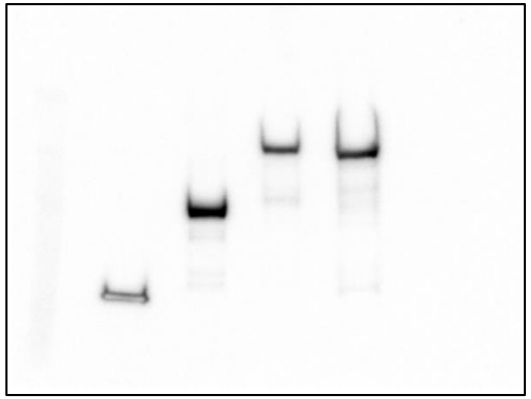

GFP

Blots from 7B.

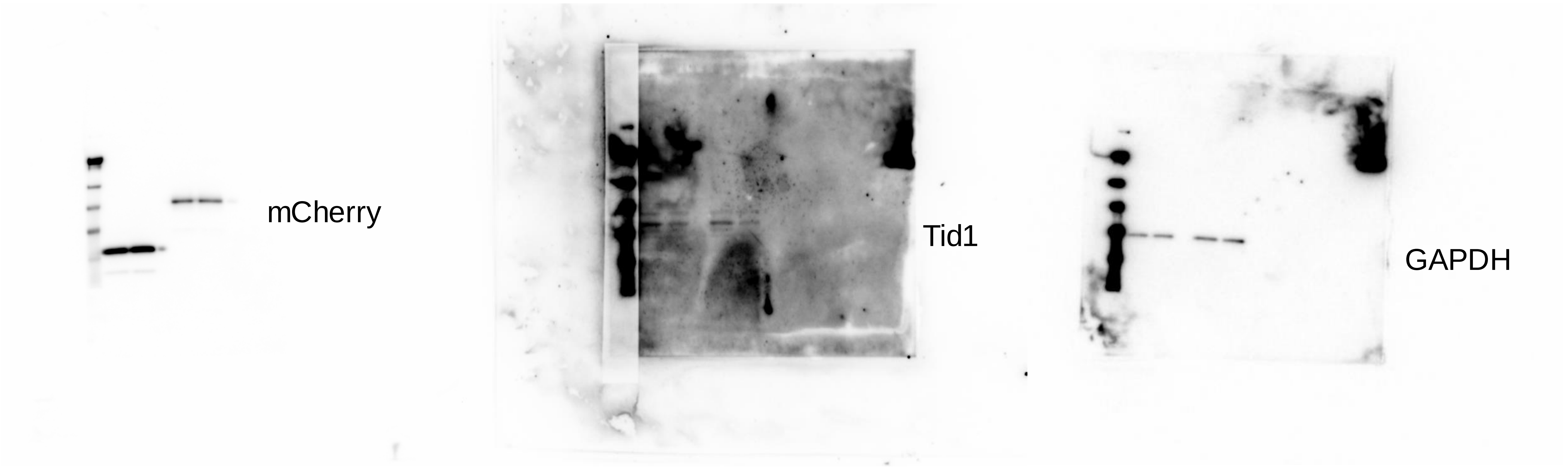

Blots from 8A

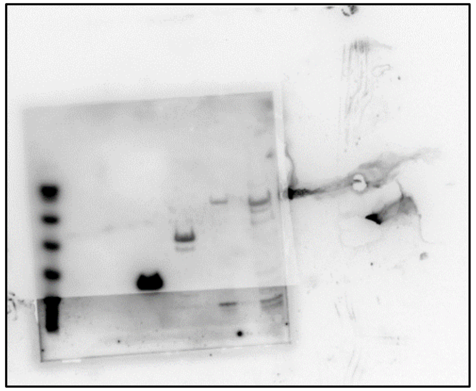

CypD

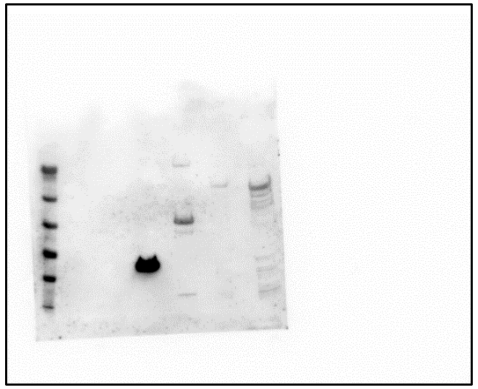

Bax

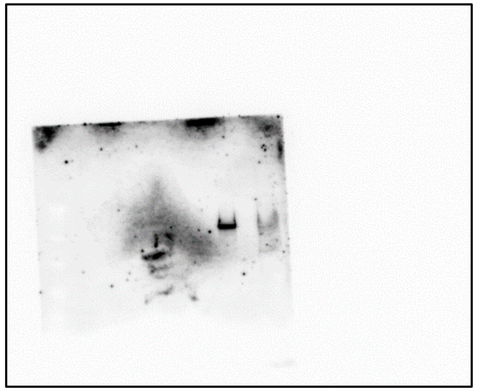

MDM2

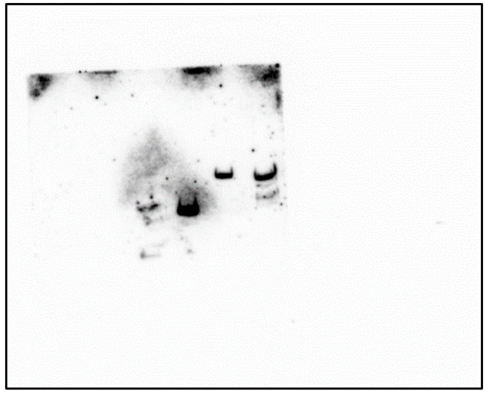

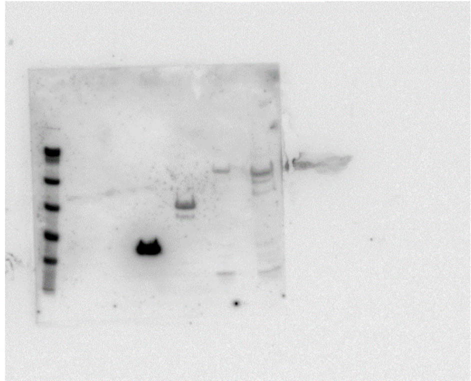

GFP

Replacement for original GFP blot to demonstrate that the lowest band (in the GFP lane) is a blown out band.

Blots from 8C.

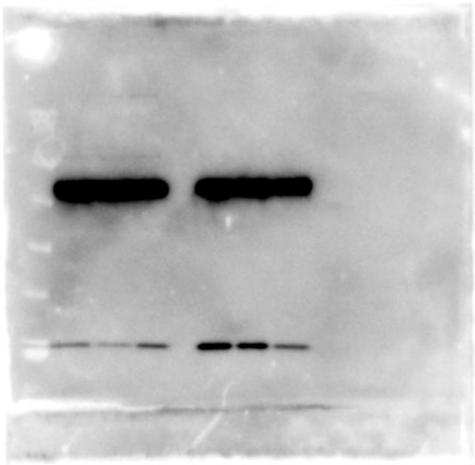

Cytochrome c

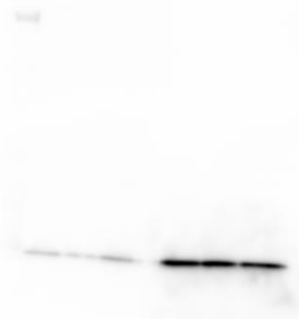

COX IV

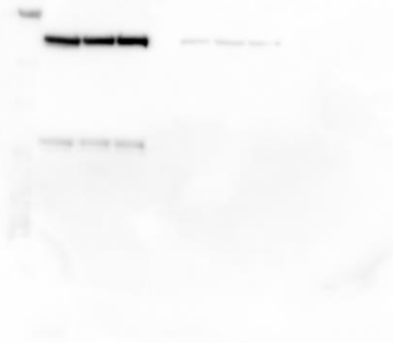

HSP90

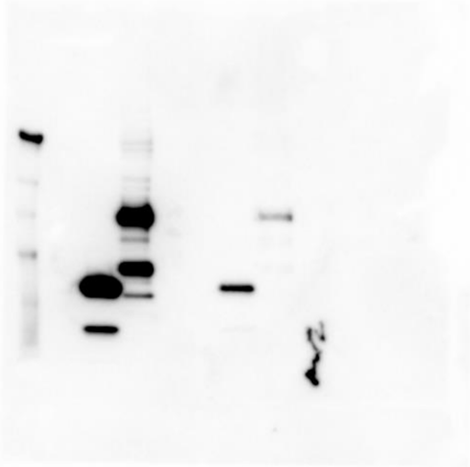

mCherry

Blots from SI2a

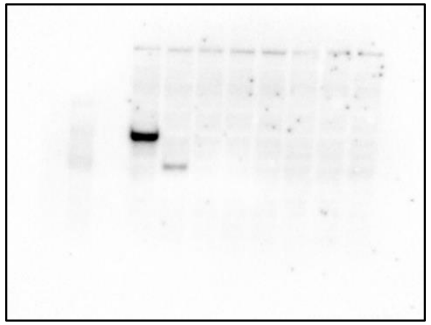

p53

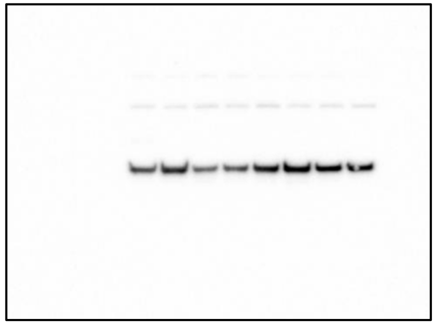

GAPDH

Blots from SI3a

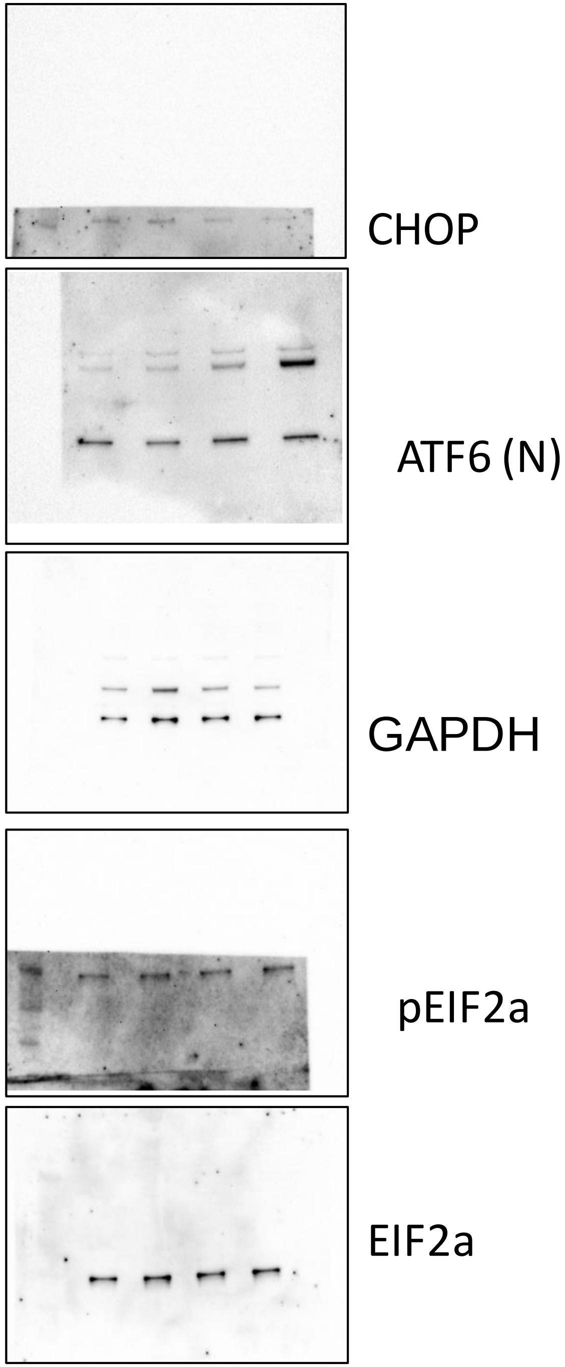

Blots from SI5a

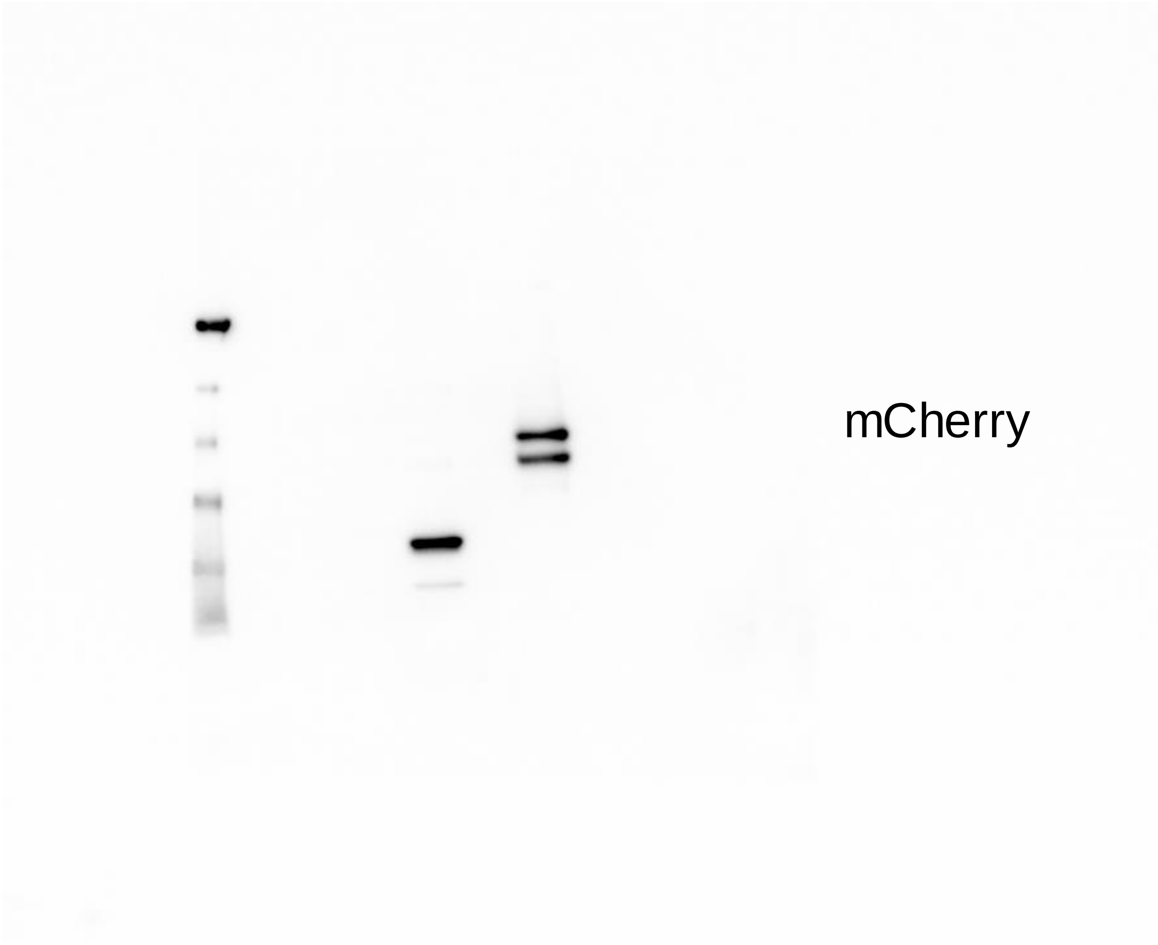

mCherry

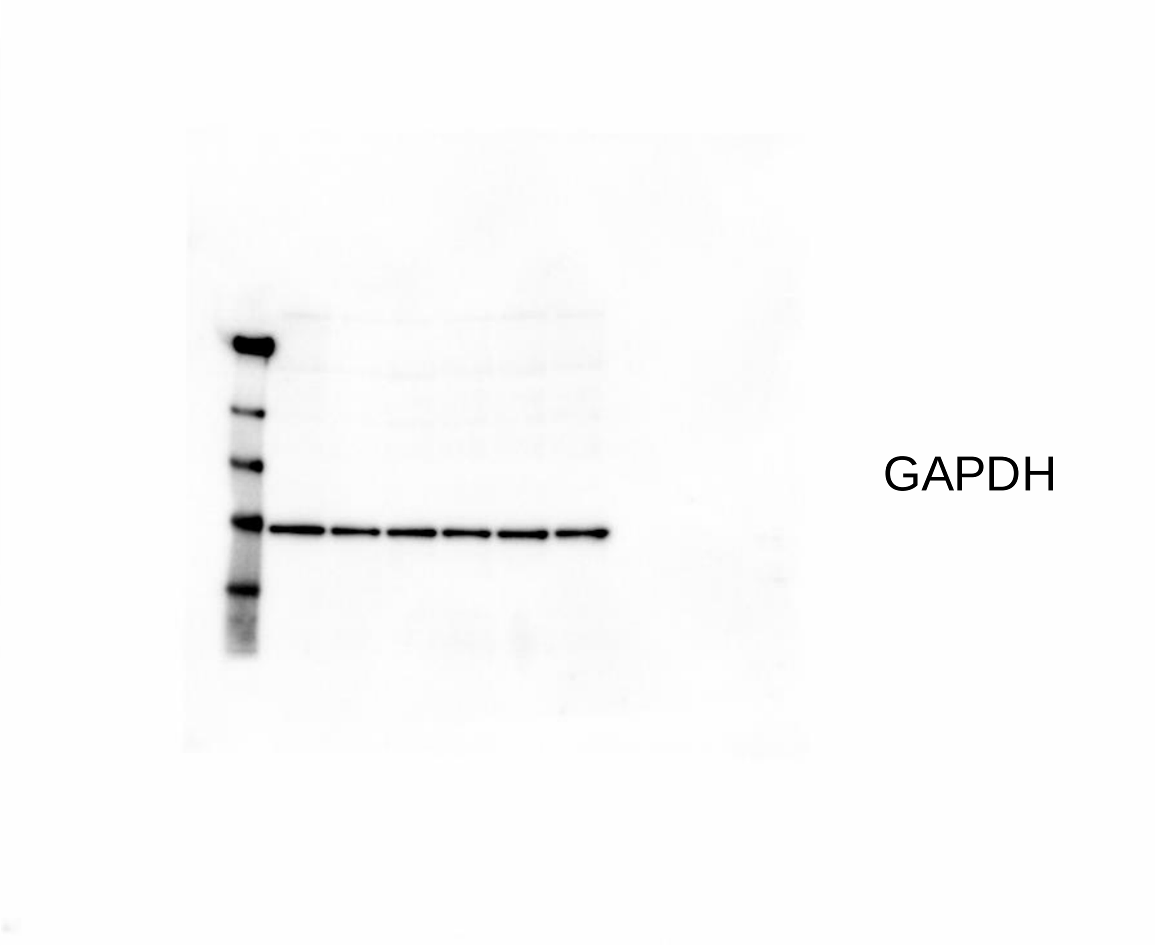

GAPDH

Blots from SI6d.

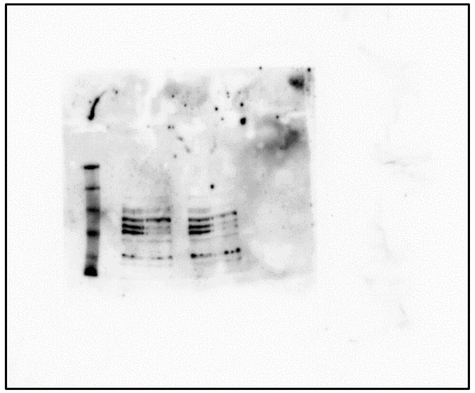

Tid1

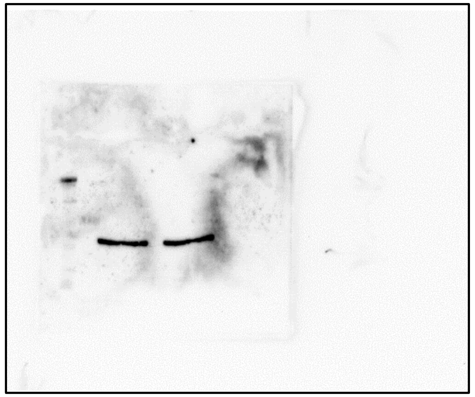

GAPDH
