# Supplementary material for: Elephant TP53-RETROGENE 9 induces transcription-independent apoptosis at the mitochondria
Source: Cell Death Discov. 2023 Feb 16;9:66. doi: 10.1038/s41420-023-01348-7 (PMC9935553; doi:10.1038/s41420-023-01348-7)
Supplement: Supplementary file 10 — Authorship change agreement [file 41420_2023_1348_MOESM10_ESM.pdf]

Aidan Preston

**From:**	Brayden Barney
**Sent:**	Thursday, January 26, 2023 11:43 AM
**To:**	Aidan Preston
**Subject:**	Re: [Ext] CDDISCOVERY-22-3686R1 Initial Quality Check

Yes I agree with the change.

Get Outlook for iOS

From: Aidan Preston <Aidan.Preston@hci.utah.edu>
Sent: Thursday, January 26, 2023 9:41:00 AM
To: Brayden Barney <u0959369@utah.edu>
Subject: FW: [Ext] CDDISCOVERY-22-3686R1 Initial Quality Check

Hi Brayden,

Could you send me an email to satisfy the following request from the journal:

3. It has come to our attention that your most recent author list differs from the one in your original submission. Please request agreement from all authors including additions and deletions, these can be collected in the following way:

Email your co-authors with the change, and ask them to reply to your email confirming that they agree to these changes. Once you have collected these replies, please combine all of the co-authors' email responses in one document and upload this file to your submission.

Thanks
Aidan

From: Cell Death Discovery <cddiscovery@springernature.com>
Sent: Thursday, January 26, 2023 4:44 AM
To: Aidan Preston <Aidan.Preston@hci.utah.edu>
Cc: Lisa Abegglen <Lisa.Abegglen@hci.utah.edu>
Subject: RE: [Ext] CDDISCOVERY-22-3686R1 Initial Quality Check

Hi Aidan,

Thank you for your email. The only changes that need to be approved by all authors are the addition of 2 authors to the author list:
Ms Rachel Anders , Mr Brayden Barney

Kind regards,
Reagan

Reagan Woon
(she/her/hers - more information why)
Editorial Assistant, Academic Journals
Springer Nature

Aidan Preston

From: RACHEL ANDERS
Sent: Sunday, November 13, 2022 2:00 PM
To: Aidan Preston
Subject: Re: Manuscript Affiliation Review

Hi Aidan,

Thanks for reaching out. My affiliation looks good to me. Hope you all are doing well in the lab. I started at the medical school here at the U now, and it's been fun seeing Josh come to lecture every once in a while.

All the best!

Rachel

From: Aidan Preston <Aidan.Preston@hci.utah.edu>
Sent: Sunday, November 13, 2022 11:42 AM
To: RACHEL ANDERS <RACHEL.ANDERS@hsc.utah.edu>
Subject: FW: Manuscript Affiliation Review

Hey Rachel,

Hoping this email is current. Tried connecting on LinkedIn too in case this does not work.

Aidan

From: Aidan Preston
Sent: Sunday, November 13, 2022 1:30 PM
To: Aaron Rogers <Aaron.Rogers@hci.utah.edu>; Miranda Sharp <Miranda.Sharp@hci.utah.edu>; Gareth Mitchell <Gareth.Mitchell@hci.utah.edu>; Cristhian Toruno <Cristhian.Toruno@hci.utah.edu>; Lauren Donovan <lndonovan11@gmail.com>; Journey Bly <Journey.Bly@hci.utah.edu>; Emily Payne <emily.payne@path.utah.edu>; Rachel Anders <Rachel.Anders@hci.utah.edu>; 'Brayden.Barney@hci.utah.edu' <Brayden.Barney@hci.utah.edu>; Joshua Schiffman <joshua.schiffman@hci.utah.edu>; Lisa Abegglen <Lisa.Abegglen@hci.utah.edu>; David Malkin <david.malkin@sickkids.ca>; 'dennisschmitt@missouristate.edu' <dennisschmitt@missouristate.edu>; 'WKiso@white-oak.org' <WKiso@white-oak.org>; cmaley1@asu.edu; biotechtwright@gmail.com; Ryan Kennington <Ryan.Kennington@hci.utah.edu>; fedak@math.utah.edu; sarah.eckstein@duke.edu; Matthew Buccilli <Matthew.Buccilli@hci.utah.edu>; 'Bahar Shamloo' <bshamloo@peeltx.com>; rosannrobinson@hotmail.com; Gabriela Furukawa <Gabriela.Furukawa@hci.utah.edu>
Cc: Lauren Donovan <Lauren.Donovan@hci.utah.edu>; Emily Payne <emily.payne@path.utah.edu>; Bahar Shamloo <Bahar.Shamloo@hci.utah.edu>; 'Rosann Robinson' <rosann.robinson@utah.edu>; Rosann Robinson <rosann.robinson@utah.edu>; Tony Iovino <tony.j.iovino@gmail.com>
Subject: Manuscript Affiliation Review

Dear co-authors,

Our manuscript was reviewed by Cell Death and Discovery, and we have worked hard over the last few months to address reviewer comments. We will resubmit next week. I've attached the edited version of the manuscript and figures
